# Measuring the impact of publicly funded open innovation programmes: the case of Data Market Services Accelerator

**DOI:** 10.12688/openreseurope.13621.1

**Published:** 2021-06-18

**Authors:** Maria Priestley, Elena Simperl, Cristina Juc, María Anguiano

**Affiliations:** 1 Department of Informatics, King's College London, London, WC2B 4BG, UK; 2 Spherik Accelerator, Bucharest, 400267, Romania; 3 ZABALA Innovation Consulting, Mutilva Alta, Navarra, 31192, Spain

**Keywords:** open innovation, data ecosystems, business accelerators, impact assessment, startups, SMEs

## Abstract

One of the current goals of the European Commission is to stimulate the development and uptake of data and AI technologies in the economy. Substantial funding has been invested in programmes that help startups and small-medium enterprises (SMEs) to assimilate the latest technical and regulatory trends. In order to assess the efficacy and impact of such initiatives, their specific social and economic objectives must be taken into consideration. Our paper proposes a generalisable mixed-methods approach for assessing the impact of publicly funded innovation programmes across multiple dimensions, including their effect on the market, fundraising capabilities of companies, innovation, and socio-economic aspects. We apply this framework to evaluate the recent performance of the Data Market Services (DMS) Accelerator, a current programme funded by the European Commission. In addition to assessing how DMS has been able to meet its objectives, our examination alerts other similar programmes of the challenges in assessing specific outcomes such as standardisation and long-term legal strategy in the fields of data innovation.

## Executive summary

This paper discusses current challenges related to the measurement of impact in open innovation programmes. Drawing on our experience from the first two years of the Data Market Services (DMS) Accelerator, we discuss some of the approaches and methods that can help to assess progress towards the specific objectives of publicly funded data incubators in Europe.

Publicly funded data innovation programmes embody a novel form of support for startups and SMEs. While these initiatives share traits in common with traditional business accelerators and incubators in terms of the services offered, public funders strive to fulfil a unique set of objectives. In addition to the financial return generated by individual firms, such initiatives have longer-term goals related to the creation of employment and regional development. Another goal particular to big data initiatives is the creation of data value chains by means of interoperable products and services. Ambitions of this kind require specialist training in legal strategy and standardisation. The substantial time and resource investment required from startups to participate in this long-term vision is not always aligned with short-term business goals and profitability. Impact assessments must therefore consider performance at the individual company and ecosystem levels, as well as deriving metrics for ex-ante analysis of wider socio-economic impact.

Based on a review of previous impact assessment approaches, we use a mixed methods framework to evaluate impact along the following dimensions:

- Market
- Funding
- Innovation
- Socio-economic aspects

Our article provides an initial assessment exercise demonstrating how this framework has been applied in practice with the DMS Accelerator, where methods such as surveys, performance metrics and workshops have been used to obtain monitoring data. Based on insights gathered from startups and programme partners over the first two years of the programme, we derive methodological recommendations and best practices.

The results of our self-assessment highlighted topical challenges that are relevant to other programmes specialising in data. We found that the DMS Accelerator showed rapid development in dimensions related to the market and fundraising, but was less impactful in some aspects of innovation and socio-economic development. Specifically, there was low interest in standardisation and long-term legal strategy among startups, as well as few opportunities for them to communicate or learn about each other’s inventions. It was therefore difficult to justify our expected impact of improved standardisation and interoperability in the context of cross-sector data applications and technology convergence. We propose that this could be addressed in future by adjusting the startup selection procedure to prioritise companies that have a stronger interest in long-term development and motivation to improve their collaboration capabilities through standardisation or Intellectual Property Rights (IPR). Additionally, a more compelling value proposition can be developed to communicate the benefits of standardisation and IPR to startups who are not yet aware of opportunities to become part of data value chains. By promoting services related to standards and legal strategy, publicly funded programmes such as DMS can help startup founders to understand the value of these practices and incorporate them into their business.

## Introduction

Many societies across the world are undergoing a transformation through technologies affiliated with AI ^1^. This industry promises to revolutionise diverse areas of economic activity, including the provision for energy, mobility, food, and healthcare. Machine-driven automation is increasingly being used by businesses to improve the efficiency of processes in logistics, productivity and customer relations. These developments rely on data as a critical asset that contributes to advances in machine learning and AI. Data-driven innovation is therefore becoming a top priority for technology strategists and policy makers in Europe and globally.

Deriving sustainable benefits from data requires a continuous supply of innovative ideas about how best to solve social, economic and environmental challenges. However, radical innovation tends to become increasingly difficult in maturing industries, as established firms guide innovation towards outcomes that have already proven to be effective. On the other hand, startups and SMEs offer the agility required to explore, generate, and deploy new radical ideas in the economy. Because smaller and newer ventures are more vulnerable to economic pressures, corporate and public stakeholders are actively directing resources into supporting and monetising the discoveries generated by them.

Support programmes for startups and SMEs have typically taken the form of accelerators and incubators. More recently, open innovation programmes have emerged at the intersection of these two paradigms by leveraging cross-organisational partnerships. In such programmes, public and private stakeholders come together to develop training, mentoring and networking opportunities that strengthen the startups’ competence in creating new products, services and business models. In the specific areas of data and AI, the support network is designed to drive new technical advances and increase productivity within firms, while at the same time helping them to monetise and exchange their data and services with external organisations. In addition to offering strategic business support, data-centred training packages address responsible data governance, standardisation and compliance with regulations. Together, the offerings of data-centered open innovation programmes help startups to introduce novel products and services into the economy, in addition to improving interoperability and trust in the AI industry more broadly.

In publicly funded programmes, the short-term economic benefits of open innovation programmes are accompanied by longer-term goals related to the creation of employment and regional development. The European Commission has partnered with the Big Data Value Association (BDVA) to build an ecosystem of projects that support the creation of new jobs, products, and services related to big data and AI in Europe. Examples of currently running programmes include Data Market Services (DMS), European Data Incubator (EDI), REACH, Media Futures, and EUHubs4Data. Programmes of this kind provide startups and SMEs with personalised coaching, training in technical and entrepreneurial skills, fundraising, and networking opportunities. They therefore serve as attractive interventions for innovation economies at the European and regional levels.

Given that public funding for these initiatives is limited, effective use of resources requires funders to know the impact of different programmes and be able to identify strategies that create the best pay-offs. The challenge is to assess the link between investing in particular support programmes and the specific socio-economic benefits achieved as a result.

The present article discusses the question of how impact can be assessed in open innovation programmes related to data and AI. Drawing on our experience of publicly-funded data incubators in Europe, we review previous approaches to impact assessment, their position in the wider economic context, and what distinguishes data and AI programmes from other types of accelerators in the European tech scene. We then propose a methodology to assess their impact and apply it to one such programme, Data Market Services (DMS), which we are currently part of. The methodology presented here is being used to monitor and adjust the governance of our services based on work done in the first two years of the programme. This paper aims to share our learning with the wider community, discuss best practices, and provide recommendations for policy makers and managers of other similar initiatives.

### Open innovation programmes for startups and SMEs

“Open innovation” is a paradigm that describes the “purposive inflows and outflows of knowledge [between organisations] to accelerate internal innovation and expand the markets for external use of innovation” ^2^. Instead of keeping the internal development of products and services within a single firm, open innovation draws upon the collaborative capacity and collective expertise of a wider variety of participants.

Applied to the areas of data and AI, open innovation programmes in Europe encourage the transfer of datasets, services, and ideas between parties in multiple ways. On one level, knowledge and resources are exchanged between the collaborative arrangement of public and private stakeholders who run these programmes. On another level, these consortia provide guidance on the infrastructures and competencies required by startups to reuse data and integrate data services with external organisations. Startups and SMEs that work with closed and sensitive datasets can benefit from secure access systems and familiarity with General Data Protection Regulation (GDPR). All kinds of digital innovation can also benefit from standardisation, where common data formats and structures enable interoperability between different data products and services. While the advantages of standardisation and compliance are recognised by the data community, in practice these activities pose a significant challenge to businesses. Startups and SMEs often need access to additional expertise and resources to implement best practices when it comes to data sharing. These kinds of support are typically included in open innovation programmes operating in data and AI.

### DMS and its mandate as part of the BDVA ecosystem

DMS Accelerator is one of the open innovation programmes that has been developed to address the major hurdling blocks faced by startups and SMEs specialising in data. Selected companies are invited to draw upon a service catalogue that helps them with fundraising, acceleration, and standards, and legal, promotion, and data skills. These services are delivered by a consortium of 10 organisations that include European accelerators, dissemination managers, standardisation bodies and universities. Overall, the programme services 150 startups and SMEs over the span of three cohorts, one per year. Unlike other programmes that offer seed funding, the DMS offer consists entirely of services that are delivered free of charge.

DMS is part of a broader ecosystem of projects associated with the Big Data Value Association (BDVA). Since 2014, the BDVA has partnered with the European Commission to support the development of jobs, products, and services around Big Data in Europe. In addition to regional development, this partnership intends to support data markets and data value chains through the transfer of data. An important source of new marketable solutions and innovations comes from the recombination of existing data sources and the collective visions of different organisations to address previously unforeseen uses. A practical challenge here relates to the collation of data from multiple sources, where interoperability requires compliance with common standards and practices. Improved standardisation and interoperability are also necessary for technology convergence and the creation of new cross-sector applications (e.g., in relation to Cloud, Internet of Things [IoT], and Privacy Preserving Technologies).

One of the difficulties in achieving these goals is that the economic benefits of standardisation may not be immediate for individual startups. Researchers have highlighted that AI companies face a trade-off between pursuing standardisation for the benefit of compatibility with other platforms versus a better fit and flexibility within their business ^3, 4^. Investment in learning and implementing common practices may offer few financial incentives for the business itself, but it reduces the burden of their clients and collaborators. For instance, standardised data formats reduce the time and effort required by an external user to incorporate the resource into their own solution, and associated services (e.g., Application Programming Interface [API]) make it possible for others to benefit from the solution without having to reinvent the same service. Together, compliance with common data standards adds value to the ecosystem by supporting long-term innovation and interoperability.

Another aspect of interoperability relates to the creation of common data spaces and common practices for sharing data between different stakeholders. The vision of pan-European data sharing spaces has been supported by the BDVA as a private counterpart of the European Commission in the Big Data Value Public-Private Partnership (BDV PPP) ^5^. Their role is to guide public and private investment towards fair, secure, and legal governance frameworks such that large-scale data can be connected and valorised. Secure data exchange and the protection of personal data are ensured through legal compliance with GDPR, as well as other European policies and directives. Industrial data platforms are additionally encouraged to adhere to EU values such as democracy, open competition, and egalitarian treatment ^5^. In order to exploit the analytic and economic value of distributed data assets within the bounds of the regulatory landscape, several innovation programmes have focused on experimentation with new business models. Initiatives such as DataPitch and the European Data Incubator (EDI) entailed ’challenges’ where startups were matched with corporate data providers to develop data-driven business solutions. Endeavours of this kind have culminated in practical learning and recommendations, with examples including the Data Sharing Toolkit and the Legal and Privacy Toolkit, which help to support data collaborations and build trust in industries affiliated with data and AI.

Despite the collective benefits of data sharing and standardisation, compliance with interoperability and ethical guidelines impose costs that may reduce the short-term revenue and productivity of individual firms. The full impact of programmes such as DMS must therefore take into account not only the financial success generated by firms who participate in these efforts, but also their actualised and potential impact on the wider entrepreneurial ecosystem. Outcomes such as the creation of jobs, data skills, collaborations, products, and reusable data sources lead to economic benefits for other stakeholders that can be monetised at future points in time.

### Different funding objectives require different impact metrics

There are various kinds of initiatives whose impact assessment approaches can be drawn upon and compared to identify relevant impact metrics for programmes such as DMS. While open innovation programmes share many characteristics in common with traditional business incubators and accelerators, they also cater to collective objectives that are more aligned with social innovation programmes. Additionally, the focus on data-driven innovation at DMS resonates with contests and datathons that have acquired a unique set of evaluation metrics. We discuss these approaches below and identify a collection of pertinent criteria to inform the design of our impact assessment methodology.

The similarity of open innovation programmes to traditional business incubators and accelerators offers an opportunity to explore how the impact of such programmes has been measured in the past. The classification of different programmes and evaluation of their impact is an ongoing challenge that has been identified in the literature ^6, 7^. Programmes differ in their business models, sources of funding, and services offered. Accordingly, numerous authors have highlighted the need for impact measures to take into account their varying objectives. Clarysse and Van Hove ^8^ identified three emerging archetypes in European accelerators: ecosystem builders (publicly funded), investors (privately funded), and matchmakers (hybrid funding). The distinction between them is summarised in Table 1.

**Table 1.** Key archetypes in accelerators. Adapted from Clarysse and Van Hove ^8^.

Archetype:	Private funding Investor-led	Hybrid funding Matchmaker	Public funding Ecosystem
Strategic focus:	Select attractive investment propositions and turn early–stage projects intoprofitable businesses.	Typically non-profit orientation. Corporates match startups to their own customers or stakeholders.	Stimulate startup activity within specific regions or technological domains.
Programme package:	Mentorship from serial entrepreneurs and business angels; often sector specific.	Coaching and mentorship from internal experts; especially helping startups to navigate corporate customers.	Mentorship from serial entrepreneurs and business developers; most developed curriculum.
Funding of startups:	Seed funding offered in exchange for equity.	Typically no seed funding.	Various funding structures and revenue models.
Selection:	Favour ventures in later stages with a proven track record.	Favour ventures in later stages with a proven track record.	Favour ventures in very early stages.
Impact assessment:	Revenue	No hard KPIs	Employment

Sources of funding introduce complexity into the identification of assessment criteria for programmes such as DMS. Traditionally, accelerators were funded by investment from business angels, venture capital funds, or corporate venture capital with the intention of capitalising on profitable startups ^8^. The return on investment was the main business model that catalysed the growth of such accelerators, and so impact was measured by the return on investment from startups. The working capital of more recent programmes has relied on shareholders such as investors, corporates and public authorities whose missions are less concerned with short-term financial gain. Instead, these programmes strive to create economic benefits on the wider entrepreneurial ecosystem and in accordance with the sustainable development goals (SDGs). 

Publicly funded innovation programmes related to data and AI typically fit across the matchmaking and ecosystem building archetypes presented in Table 1. Examples of past programmes include ODINE, DataPitch, STARTS, and Future Internet (e.g., FINODEX). Current programmes include DMS, REACH, Media Futures, and EUHubs4Data. 

Previous systematic studies into the performance of different accelerator models have relied on metrics such as the funding raised and valuation attained by the companies, revealing that graduates of publicly sponsored programs tend to raise significantly lower sums of capital post-accelerator ^9^. Clarysse and Van Hove ^8^ highlight that accelerators financed under the objectives of regional development and employment struggle to be profitable in the short or even medium term. These programmes’ selection criteria and success in meeting socio-economic objectives are not aligned to the creation of profit.

While previous publicly-funded programmes have nonetheless monitored firm-level financial performance as part of their impact assessment, many have also traced the impact on employment, society, and the environment. This has been particularly true of social innovation programmes such as those from the past EU call for “Collective Awareness Platforms for Sustainability and Social Innovation” (CAPS). Within this call, the Impact Assessment for Social Innovation (IA4SI) project defined a mixed methods framework encompassing social, economic, political, and environmental impacts ^10^. Although this example offers a comprehensive methodological structure, it would not fully reflect the focal intention of DMS in terms of technological and data-driven entrepreneurship.

Initiatives that have a stronger focus on data innovation offer insight into some of the additional metrics that may prove useful. In particular, hackathons and short innovation contests hosted by platforms such as InnoCentive, TopCoder, and Kaggle provide well-defined challenges where data scientists, researchers, and developers compete to solve complex problems presented by industry or public stakeholders. Such experiments have contributed to the procurement of novel solutions in fields such as health, criminology, and search technology. The impact of innovation contests has been assessed by frameworks such as the ICAPT (Innovation Contests as an Alternative Procurement Tool), which compares the cost of solutions procured through contests relative to the estimated cost of developing an equivalent solution by traditional methods ^11^. This framework also evaluates qualitative benefits such as project awareness and best practices for management. It has been found that contests are cheaper ^12^, and that their collaborative format enables solutions to be discovered much quicker and through a more diverse range of participants ^13^. These past findings imply the utility of impact metrics that assess the cost, speed, and diversity of innovation contests and research experimentation.

The discussion above drew on a range of innovation initiatives whose impact assessment criteria can be relevant to programmes such as DMS. However, we are faced with the challenge of evaluating a programme that sits at the intersection of financial, social, and technical objectives. The desire to support the profitability of individual firms is combined here with a vision for sustainable long-term development and technological innovation, requiring a unified impact assessment framework. There are a number of past and current programmes that tackled similar transversal objectives in their impact assessments:

- **Open Data Incubator Europe (ODINE) ^1^** was a programme for open data entrepreneurs that reported impact in terms of the number of incubated ideas, return on investment, engagement, jobs, and geographic representation.
- **Data Pitch** matched data providers with startups who worked to address open innovation challenges. Their impact assessment reported on the number of data-driven businesses established, cross-sector and cross-border collaborations, financial impact, and the creation of big-data use cases that drive investment ^14^. Following its completion, the programme organisers developed additional resources that could be used by other organisations. For example, the Data Sharing Toolkit helps organisations to generate value by allowing third parties specifically permissioned access to private datasets.
- **Science, Technology and the Arts (STARTS)** was a residential innovation programme designed to increase the impact of artists in high-tech scientific environments ^15^. They committed to deliver a certain number of residencies, a global methodology and tools to promote collaborative work, as well as knowledge to evaluate success factors in future initiatives.
- **Future Internet (FI)** encompassed a number of accelerators working in Internet-enabled innovation ^16^. The project developed analytic methods and tools to perform an ex-ante socio-economic impact analysis. Their framework contained several assessment areas and KPIs including:
**Market** - customers, revenue, and geographies (footprint in EU economy)
**Socio-economic** - social, scientific, and macro-economic consequences (e.g., wider perception of AI)
**Innovation** - types of technology solution, intellectual property (IPR)
**Funding** - funding requested and quality of financial plan

- **DataBench** is currently creating a benchmarking process for organisations that develop Big Data Technologies (BDT) ^17^. The framework measures technology development activity against parameters of high business relevance, seeking to demonstrate industrial significance and return on investment.

### Methodological implications

What unites the programmes listed in the previous section is that their impact assessment approaches drew on a variety of metrics to capture financial, social, and technological outcomes. Additionally, they presented nuanced methodological considerations in terms of benchmarking and assessing qualitative impacts. In the following paragraphs we discuss what the quantitative and qualitative impacts commonly entail, and suggest ways of synthesising these strengths into a generalisable impact assessment approach.

When it comes to quantitative metrics, a number of earlier programmes have been able to conduct rigorous evaluations of impact through counterfactual analysis. For example, Data Pitch compared their outcomes with what would have been achieved by startups in the absence of the programme ^14^. This kind of analysis is conceptually similar to benchmarking approaches such as Data Bench, where the impact assessment is based on relative comparisons. However, it is important to consider that not every programme will have the resources to benefit from counterfactual analysis or benchmarking tools. In the present case, we propose the comparison of measurements across multiple cohorts of the same programme as a simpler way to assess impact in terms of temporal improvement and agility in the specific attributes relevant to that programme.

When evaluating qualitative impacts, previous data innovation programmes were alike in reporting on their methodological contributions and learning. Publicly funded accelerators are characterised by high quality self-assessment and transparent reporting of outcomes, often sharing expertise that has an impact on the wider BDVA ecosystem. Due to the limited duration of publicly-funded programmes (DMS lasts three years) it is not usually possible to measure the actualised impacts and value chains that accrue over the longer term. However, resources that have been released into the ecosystem for future use are one of the ways of ensuring lasting impact. We therefore propose viewing methodological contributions as one of the manifestations of socio-economic impact. In the case of the DMS Accelerator, one of the defining qualities of the programme is that it attracts startups based purely on its services, without offering seed funding. The methods that were developed for selecting and onboarding startups who are motivated and engaged by this service proposition could therefore be an impactful resource for other accelerators whose funding model is similar to that of DMS.

In addition to revealing impact assessment approaches that are held in common by multiple programmes, our overview also highlighted challenges for developing a unified impact assessment methodology. Some past programmes such as Data Pitch were structured in such a way that startups were systematically matched with data providers, making it possible to demonstrate well-defined data experiments and collaborations at the broader socio-technical level. However, because this indicator relied on the specific format of the programme, it would be difficult to replicate the same assessment in another programme such as DMS. Our intention in this article is to identify a flexible and generalisable framework, which is applicable to DMS but not endogenous to it.

We suggest that the main impact categories defined previously by the FI-Impact project can be used as the basis for such a framework. Specifically, the categories of 1) Market, 2) Funding, 3) Innovation, and 4) Socio-economic aspects provide a comprehensive yet generalisable representation of the main areas of impact applicable across publicly funded data innovation programmes. In addition to assessing impact on the entrepreneurial ecosystem in terms of sales and investment, the dimensions also accommodate technical advances and qualitative socio-economic impacts. For the purposes of flexibility, the specific metrics within each impact category can be defined in accordance with the monitoring opportunities of particular programmes. A variety of qualitative and quantitative methods can be combined to assess each impact dimension during successive years of the programme. In the remainder of this article, we demonstrate how the four-dimensional impact framework has been implemented in practice at DMS Accelerator, and the results gained from our experience.

## Methods

The DMS programme is unique in its focus on service provision, rather than pre-defined data challenges or collaborations that have characterised other similar programmes in the past. Our methodological framework therefore has an emphasis on the evaluation of services (in terms of engagement and satisfaction), while at the same time incorporating financial successes of firms, new products, collaborations, and public awareness metrics. DMS services are already classified into categories (Acceleration, Promotion, Fundraising, Standards and Legal, and Data Skills) that lend themselves to the FI-Impact dimensions (Market, Funding, and Innovation). Moreover, the expected impacts of the DMS programme that are outlined in the grant agreement also map onto all dimensions, including Socio-Economic impact. These expected impacts are presented in Table 2 (first column).

**Table 2.** Impact assessment methodology proposed for DMS.

Dimension Followed by expected and additional impacts as specified in the DMS grant agreement	Individual company metrics	Collective metrics / KPIs
**Market** DMS expected impact: • At least 50 clients (e.g., start-ups, SMEs) served annually in partner finding, matchmaking, venture capital raising, training, and coaching • Reach 2,000 companies out of which 150 will be serviced • Increase their sales capacity by 15%	• Sales capacity • Revenue • New clients	• Number of contacted and recruited companies • Companies at different stages of development • Representation of different industry sectors and countries • Engagement in acceleration webinars
**Funding** DMS additional impact: • New rounds of private capital reaching 5m euros • Additional public funds reaching 1m • Capacity for future investment and partnerships offered by the consortium and Advisory Board of investors	• Additional funding gained	• Engagement in fundraising webinars • Meetings with investors
**Innovation** DMS expected impact: • Offer training on standards and legal issues to 150 companies • Improved standardisation and interoperability especially in the context of cross-sector applications and technology convergence (data, Cloud, IoT, connectivity)	• New products, datasets, services • Patents	• Engagement in standards & legal webinars • Engagement in data skills courses • Mentoring sessions in standards & legal
**Socio-economic** DMS expected impact: • Success stories as a result of services offered • Dissemination and exposure of success stories DMS additional impact: • At least 200 new jobs requiring data skills will be created in the portfolio of companies	• Jobs created • Gender composition of teams • Collaborations • Success stories	• Social media followers • Website visitors • Audience at events • Self-organised events • Methodology shared with other programmes
Method of assessment:	Surveys supplemented with desk research	Programme monitoring metrics and workshops with programme partners

The four-dimensional impact criteria address the tension between the short-term and long-term economic impacts of data innovation programmes. At DMS, this is achieved through metrics that monitor impact at two levels of analysis: 1) the success of companies who complete the programme, and 2) impact on the collective ecosystem (the entrepreneurial environment as well as on other open innovation programmes). We evaluate these outcomes using a mixed-methods approach. Quantitative metrics are derived from programme monitoring activities and close-ended survey questions completed by startups when they leave the project. Qualitative approaches were additionally used to gain a more nuanced understanding of these metrics. For example, open-ended survey questions were used to assess the ways, if any, that participation in DMS contributed to the successes of startups in specific dimensions. We also conducted workshops with key partners from DMS to examine the collective impact of the programme and observations that were not covered by routinely monitored KPIs.

The results presented in this article are based on programme monitoring metrics, surveys completed by startups, and workshops with DMS partners. Ethical considerations surrounding these data sources are discussed below, followed by descriptions of how each type of data was gathered.

**Ethics statement** Our investigation reuses data that were collected by the DMS Accelerator as part of its routine monitoring activities. The data are fully anonymised and we have obtained verbal consent from the DMS partners for our secondary use of the data. When collecting information, DMS informs participating companies that the gathered data may be used for research and reporting purposes. Additional consent to openly publish the data was not obtained due to the inclusion of commercially sensitive information that needs to remain private as outlined in the data availability statement. As our study is low risk and does not use personal data, approval from the King’s College London ethics committee was not required. Our study was done in consultation with the Information Compliance team at King’s College London.

**Routine monitoring metrics** were sourced from records kept throughout the first two years of the programme, where available. This includes engagement and dissemination KPIs reported by DMS in its periodic report from the year 2020, as well as webinar and course engagement statistics. Our analysis includes all data that fall within the scope of the expected and additional impacts presented in the DMS grant agreement, as listed in the first column of Table 2. For the purposes of brevity, we have excluded information relating to specific events and webinars conducted by DMS. Instead, the metrics are reported here in aggregate form to reflect the average attendance and satisfaction in each service category.

**Survey responses** were gathered using an ’Impact Survey’ delivered to the first two cohorts of graduates from the DMS programme. Blank templates of this survey are available as described in the *Data Availability* statement ^18^. The survey contained a selection of multiple-choice, closed-ended, and open-ended questions related to topics that are summarised in the middle column of Table 2. The closed-ended questions were compulsory, while the open-ended questions that followed them were optional. Participants who did not provide written answers for a particular question were not included in the results reported for that question. The questions were slightly different in each DMS cohort in order to account for differences in the amount of time that had elapsed since they completed the programme. Surveys to both cohorts were distributed by the coordinator of DMS Accelerator in November 2020, using an email that invited the companies to submit their responses in a Google Form. A reminder email was sent in December 2020 and DMS mentors encouraged companies to respond where possible. The response rate was 12% (six out of 50) from cohort one and 42% (20 out of 50) from cohort two. Due to the temporal setup of the DMS Accelerator programme, the responses of cohort one reflect outcomes that were achieved approximately 11 months after the startups left the programme, whereas the responses of Cohort two reflect their immediate outcomes.

**Workshop outcomes** are based on two two-hour workshops conducted with DMS partners. One of these was the Interim workshop which took place on 10th December 2019, in Leipzig, Germany. This was attended by 13 participants from nine partner organisations who had already been invited to be part of the DMS Consortium meeting on the same date. The workshop used a structure that alternated between small group discussions and plenary discussions, forming three 30 minute blocks where participants discussed topics related to startup selection, service provision, and acceleration. Participants were provided with prompts inviting them to comment on what went well, what could have gone better, and what could be done to improve the programme. The results of this workshop were summarised in a confidential Workshop Report deliverable at DMS. Subsequent actions were also taken by the relevant DMS partners to modify the programme’s startup selection and acceleration process in accordance with the recommendations raised by the consortium. In the second year, DMS organised a White Paper workshop on 12th November 2020. 11 participants attended from seven DMS partner organisations, who were invited to participate in a Microsoft Teams meeting. As with the previous workshop, this setup alternated between small group discussions in breakout rooms and joint plenary discussions. There were three 30 minute blocks where participants discussed the most and least successful aspects of the programme, how impact should be defined and assessed, and ways to make the programme more successful in future. The outcomes of this workshop informed the design of the DMS Impact Survey and the content of this paper. A summary of the structure of both workshops is available in the *Extended Data* repository ^18^.

## Results

Our findings are grouped into sections according to the four dimensions of the impact assessment framework: Market, Funding, Innovation, and Socio-economic aspects. The results for each dimension are a triangulation of the methods described above, in the form of monitoring activities, Impact Surveys completed by startups and workshop participation from programme partners.

### Market

**Figure 1.**
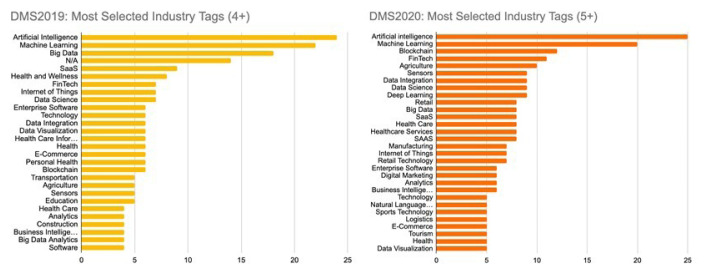
DMS applications in 2019 (left) and 2020 (right) classified by industry tags.

In the dimension of the market, our methodology sought to measure the footprint created by the DMS programme on the EU economy. As part of this, we examined the diversity of companies serviced by the project, their engagement with entrepreneurial training and their own capacity to serve new clients. Our assessment of market impact is summarised in Table 3. There are a number of ways through which DMS ensures market diversity, and these are part of the routinely monitored KPIs. The first intention is to purposefully accept companies from different stages of development, with 15 scaling, 30 validating, and five establishing companies selected each year. Between the first and second year of the programme, an increase was achieved in the pool of contacted companies, the number of countries represented in the selected portfolio and the representation of AI, ML, and other industry sectors ( Figure 1). These findings suggest an improvement in the geographic and industrial coverage of the programme.

**Table 3.** Assessment of market impact.

	Cohort One	Cohort Two
**Impact Survey** ** answers from ** **startups**	(six responses, surveyed 11 months later) • Sales capacity – 50% reported an increase in sales capacity. The average increase was 60%. • Revenue – 66.7% reported an increase in revenue. The average increase was 55%. • New clients – 33% of respondents acquired potential new clients directly as a result of DMS. Open-ended answers indicated that the effect of DMS on market impact was achieved by means of improved public image (e.g., through promotional videos and improved negotiation capacity).	(20 responses, surveyed immediately) • Sales capacity – 45% reported an increase in sales capacity. The average increase was 42%. • Revenue was not included in the survey, as we did not expect an immediate impact. • New clients – 15% gained new clients as a result of joining DMS, with five new clients on average. Open-ended answers mentioned improved company profile and marketing, increased reach and partnerships, improvement in knowledge, and selling proposition.
**Programme ** **Monitoring**	• Contacted companies: 690 • Company stages: 15 scaling, 30 validating, five establishing • Company stages: 15 scaling, 30 validating, five establishing • Countries represented in portfolio: 16 • 31 mentoring sessions for 10 startups Participation in webinars: • 10 entrepreneurial webinars, five participants on average • Two promotional webinars, three participants on average	• Contacted companies: 1,172 • Company stages: 15 scaling, 30 validating, five establishing • Countries represented in portfolio: 20 • 61 mentoring sessions for 23 startups Participation in webinars: • 11 entrepreneurial webinars, 24 participants on average, average rating 4.17/5 • Three promotional webinars, 14 participants on average, average rating 4.15/5
**Workshops with** ** partners**	DMS partners felt that the selection process was successful in attracting a diverse range of companies, representing a strong portion of the EU market. However, the service offering was not communicated clearly in relation to the needs and business models of startups. Low participation in webinars was flagged as an important challenge to address.	Based on feedback after cohort one, DMS improved its communication of services and onboarding process for startups, leading to increased engagement with all training. More personalised mentoring was provided. The market impact of startups also benefited from corporate videos created by DMS.

Over the span of two years, programme monitoring and workshop activities highlighted a dramatic increase in the level of engagement with promotional and entrepreneurial services. For example, the number of webinar participants in the Acceleration category increased fivefold from cohort one to cohort two. While it is difficult to assess the extent to which these and other DMS services directly influenced the market success of companies, the results of the Impact Survey from both cohorts indicate that twelve (approximately 44% of all respondents) saw an increase in sales capacity. In the optional question that followed, seven of these respondents provided a quantitative estimate of their increase in sales capacity, amounting to an average increase of 47% between them. A further three respondents mentioned qualitative improvements in general process development, product development and pilot studies. Besides sales capacity, 19% of respondents reported gaining new clients directly as a result of DMS. Open-ended survey answers reported that the training and promotional services offered by DMS helped to improve their company image and reach. Workshops with partners reflected these comments and established that one of the main sources of such impact came from professional videos that were created for the start-ups, who were then able to publish them on their own social media or website.

Together, the findings show good progress towards meeting the expected market impacts of the programme, especially in KPIs related to the diversity of the audience and the number of startups served. The target of increasing startups’ sales capacity by 15% has been more difficult to assess due to low survey response rates as shown in Table 3. Among those who responded, there appears to be a split where half of the companies saw no increase at all, while others reported a substantial increase. This difference in outcomes is consistent with the results observed in other accelerators, whose overall impact can be traced to a much smaller portion of serviced companies that become highly successful ^9^.

### Funding

In the dimension of funding, we assessed the amount of funding generated by DMS startups since starting the programme. We were also interested in the ways in which DMS services contributed to this success. Our assessment of fundraising impact is summarised in Table 4.

**Table 4.** Assessment of fundraising impact.

	Cohort One	Cohort Two
**Impact Survey** ** answers from** ** startups**	(six responses, surveyed 11 months later) • 33.3% gained additional funding, collectively reporting 730K Euro (unclear whether public or private) • Further desk research showed that more than 5M in private and more than 600K in public funding had been raised overall. Open-ended survey answers indicated that startups’ fundraising activities benefited from getting acquainted with DMS accelerators, learning about IP protection, and receiving promotional videos.	(20 responses, surveyed immediately) • 30% gained additional funding. 850K private and 280K public. Open-ended answers indicated that the startups gained confidence in pitching and acquired a better understanding of funding opportunities. The Pitch Day and TNW feature also initiated some introductions for them.
**Programme ** **Monitoring**	• 40 meetings facilitated with investors • Seven fundraising webinars, five participants on average	• 41 meetings facilitated with investors • Seven fundraising webinars, 18 participants on average, average rating 4.28/5
**Workshops with** ** partners**	DMS partners reported that investor pitch decks and matchmaking strategies could be more personalised and tailored to the needs of the beneficiaries.	Fundraising went well this year. A high number of meetings with investors was facilitated and clear startup success stories identified by DMS mentors.

While the programme has no concrete KPIs related to fundraising among startups, several desired impacts were included in the grant agreement. Specifically, DMS aimed for its startups to raise new rounds of private capital reaching 5meuros and additional public funds reaching 1m. Capacity for these investments and partnerships was supported by the consortium and Advisory Board of investors.

Impact assessments conducted using surveys suggested that 280K of public and 850K private funding had been raised by 30% of respondents from cohort two by the time they left the programme. In cohort one, 33.3% of respondents had succeeded in securing funding, collectively generating approximately 730K Euro within 11 months. Based on the available data, the proportions of successful DMS graduates seems to be roughly consistent with meta-analyses US accelerators, where 23% of companies were found to be successful in raising significant funds after completing the programme ^9^.

Due to the low survey response rates, the topic of funding was investigated with additional desk research on cohort one, which revealed that more than 5M in private funding and more than 600K in public funding had been raised in reality. When asked about the ways in which DMS contributed to their fundraising activity, survey respondents commented on their improved confidence in pitching and a better understanding of funding opportunities, as well as their improved image developed through the promotional services of DMS. Alongside this, programme monitoring statistics showed a growing engagement with services related to fundraising, where the number of participants in fundraising webinars had tripled in the second year of the programme. Through workshops with DMS partners, we also learnt that programme mentors provided personalised guidance and facilitated additional meetings with investors, which contributed to the acquisition of funds and identification of clear success stories among startups.

### Innovation

In the dimension of innovation, our methodology sought to capture novel technology solutions and intellectual property as indicators of impact. This dimension encompassed the role of data regulations, legal strategy, standardisation, and interoperability in supporting data value chains. Our assessment of innovation impact is summarised in Table 5.

**Table 5.** Assessment of innovation impact.

	Cohort One	Cohort Two
**Impact Survey** ** answers from ** **startups**	(six responses, surveyed 11 months later) • New products or services developed by 16.6% of startups Open-ended answers indicated that startups improved their GDPR compliance and put data management at the core of their business after leaving DMS.	(20 responses, surveyed immediately) • New products or services developed by 50% of startups Open-ended answers reported that DMS helped startups with GDPR compliance, product development strategy and data science skills.
**Programme ** **Monitoring**	• Eight mentoring sessions in standards and legal requested by seven startups. • Three Data Skills courses, seven participants on average • Seven Standards & Legal webinars, four participants on average	• 10+ mentoring sessions in standards and legal • Three Data Skills courses, 16 participants average • Seven Standards & Legal webinars, nine participants on average, 3.8/5 average rating
**Workshops** ** with partners**	New services were proposed in relation to intellectual property rights (IPR) and IP mentoring.	Mentoring sessions on GDPR and IPR received excellent feedback. However, delivery was constrained by limited resources and the scope of these services was not clearly distinguished from consultancy. Low commitment and awareness of standardisation among SMEs made it difficult to engage them with services related to standards.

DMS fulfilled its expected impact of delivering training on standards and legal issues related to data. Programme monitoring activities showed that engagement with Data Skills courses had doubled in the span of two years. Survey responses from startups also indicated that the portion of startups who developed new products or services had tripled in the second year. These products included web services, mobile and cloud platforms for various sectors, demonstrating potential alignment with the DMS expected impact of fostering cross-sector applications, and technology convergence. In their written answers, the survey respondents reported benefiting from DMS services related to compliance with GDPR and product development strategy. This feedback was reflected in the comments shared by DMS partners during workshops, where they reported that mentoring sessions on GDPR and IPR received excellent feedback.

In addition to positive feedback, workshops with partners revealed a number of limitations related to the uptake of standards and legal services. While mentoring in GDPR was in high demand, other DMS partners observed a low commitment and awareness of topics related to standardisation and long-term legal strategy (e.g,. intellectual property) among startups. Although services related to standards and legal issues experienced an increase of interest between cohorts, this service category had much lower participation compared to entrepreneurial and fundraising training.

### Socio-economic aspects

In the socio-economic dimension, our methodology sought to measure the direct and indirect consequences of DMS in terms of societal and macro-economic forces. The impacts considered here include employment and the capacity to improve the wider perception of AI among European citizens. Our assessment of socio-economic impact is summarised in Table 6.

**Table 6.** Assessment of socio-economic impact.

	Cohort One	Cohort Two
**Impact Survey** ** answers from ** **startups**	(six responses, surveyed 11 months later) • Jobs created – 50% of startups reported that their team has grown, with three new jobs created on average. Two of these respondents did not detail which roles were created, while the remaining respondent specified that they hired one business developer. • Gender composition of teams – 7% reduction in women in one startup • Collaborations – 66.7% pursued new collaborations or partnerships, helped by DMS promotion and networking.	(20 responses, surveyed immediately) • Jobs created – 50% of startups reported that their team has grown, with three new jobs created on average. Half of these respondents specified the nature of the roles, with technical and data positions being most prevalent. • Gender composition of teams – 30% reported a change, 55% of which reported an increase in the proportion of women. • Collaborations – 90% pursued new collaborations, with DMS having helped to reach new audiences and markets. Open-ended answers gave mixed feedback, with comments of needing more personalised support with connecting to investors and more interaction between startups.
**Programme** ** Monitoring**	• Social media followers: 879 • Website visitors per month: 484 • Audience at events: 1,600 (Pixels Camp) & 15,492 (TNW) • Self-organised events: 11	[pending engagement metrics] Methodology shared with other programmes: • Form for engagement with companies • Impact assessment approach (this paper)
**Workshops** ** with partners**	Mentoring sessions, bootcamps and events provided opportunities for startups to interact directly with the beneficiaries of the programme, and this was positively appreciated on both ends.	Promotional campaign of the programme was good, but few opportunities were available for communication between startups. Visibility of the project across the European ecosystem could also be improved.

Responses to the Impact Survey showed that around half of the serviced companies had created new jobs within the duration of the programme or shortly after. On average, three new jobs were created by each positive respondent and these jobs were related mostly to software development and data skills. While this contributes towards the DMS desired goal of creating 200 new jobs requiring data skills, the magnitude of progress towards that number is difficult to assess due to the low survey response rates from startups after completing the programme.

DMS monitors additional social impacts such as the gender composition of startup teams, their collaborations, and published success stories. Impact Surveys showed that, compared to the first cohort, each of these areas demonstrated an improvement in the second cohort. While the proportion of women in the workforce showed a relatively small increase, 90% of startups demonstrated a positive socio-economic impact in the pursuit of new collaborations. These startups reported benefiting from the networking opportunities provided by DMS, but they also requested more personalised support in regards to networking with investors and interacting with other DMS startups. The latter point was also highlighted by DMS partners during workshops, where communication opportunities between startups were identified as a weakness in the current service offering.

At the collective level of analysis, programme monitoring statistics indicated a good promotional campaign, presence at events and a large audience. Through feedback acquired from mentors and Impact Surveys, a number of success stories were identified among startups who benefited from the programme. The dissemination of these stories was increased through the promotional reach of the programme.

In addition to the social impact of DMS in terms of public engagement, we were also interested in assessing its position in relation to other similar programmes. Workshops with DMS partners revealed that there was room to increase our visibility and create closer partnerships with other open innovation programmes in Europe, especially on regional and local levels. In addition to approaching these connections directly, the outputs of the programme could be used to gain exposure. In particular, deliverables such as the startup selection and onboarding procedure in cohort two ( DMS deliverable D2.3) and this paper are publicly available, such that other similar programmes can benefit from relevant parts of our methodology.

### Alignment with startup needs

In the second cohort of the programme, DMS used a survey to assess the needs of participating startups before they entered the services. Startups were asked to rank different service categories from those that were most important to the least important. In order to assess the extent to which the subsequently received DMS services aligned with these needs, we included a question in the Impact Survey where startups were asked to rank which of the same business dimensions had benefited most from completing the programme.

**Figure 2.**
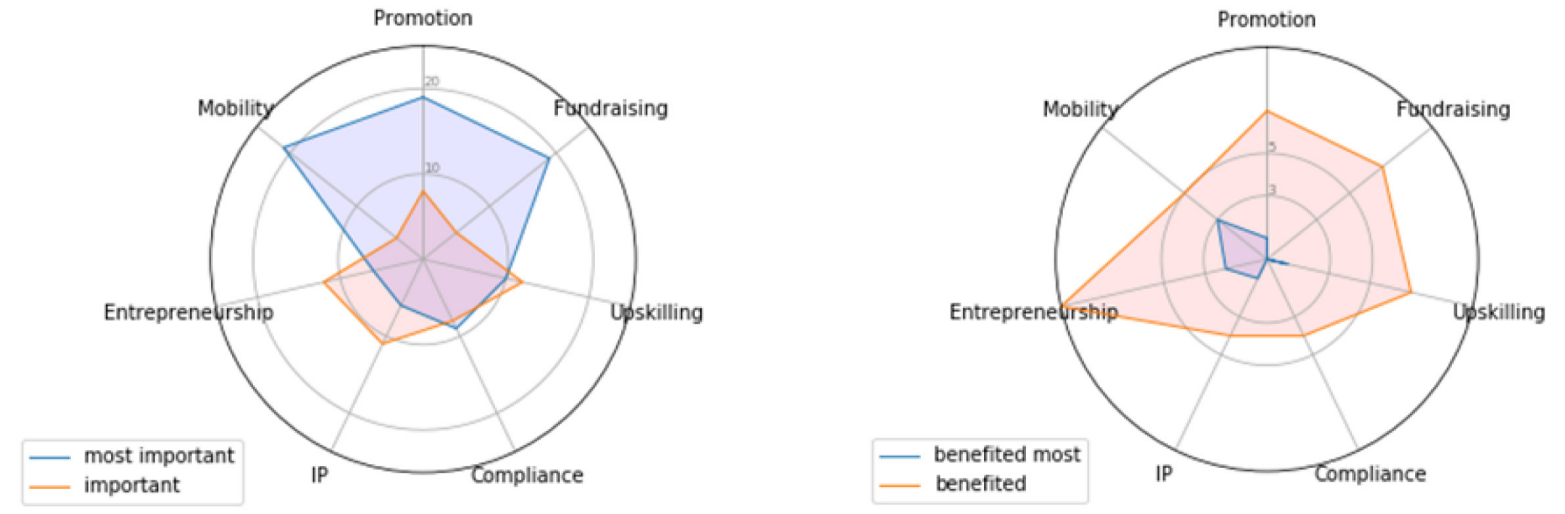
Radar charts showing the desired (left) and actualised (right) benefits among startups who participated in DMS.

Figure 2 shows the outcomes of this analysis. The radar chart on the left shows the distribution of the two most important needs identified by startups in the Needs Survey (N=47). This chart shows which needs were rated as highest (blue) and second highest (orange) by each startup, with the axis measuring the total number of participants who valued each dimension. The chart on the right shows the distribution of the areas that had benefited most from participating in DMS according to the Impact Survey respondents (N=20). By looking at these results, we see that startups went into the programme wishing mainly to develop their skills in international markets, promotion, and fundraising. However, by the end of the programme, their business acquired a more rounded range of benefits that were distributed more equitably across all areas, including entrepreneurship, IP, and data skills.

## Discussion

Building on prior literature and impact assessment methods in previous open innovation programmes, this article presented a general methodological framework and applied it in the context DMS Accelerator. We have demonstrated how dimensions related to the market, fundraising, innovation and socio-economic aspects can be assessed through a combination of qualitative and quantitative indicators.

Our results show that DMS showed a positive impact in every evaluated dimension, but that the magnitude of impact was different in each area. The programme was most successful in meeting its expected impacts in relation to the market and fundraising. However, some aspects of innovation and socio-economic impacts were more challenging to assess. We summarise these findings below and present recommendations for other similar programmes.

### Areas of greatest impact

In relation to the market, monitoring data showed high diversity in the developmental stages, geographic origins and industry sectors of companies serviced by DMS. The impact of market training was demonstrated by high engagement with entrepreneurial services and direct impacts on the sales capacity and client base reported by graduating firms. Together, these findings give confidence that DMS has improved the entrepreneurial capacity of various regions in Europe.

In the dimension of fundraising, DMS was able to demonstrate impressive successes in the number and size of investments generated by graduating firms. Monitoring data from surveys showed that at least some of these successes were attributed directly to the training and guidance that had been acquired by startups through DMS ^18^. It is worth noting that despite having objectives that extend beyond financial success, the fundraising abilities of DMS graduates were comparable to those of profit-driven accelerators.

It was more challenging to trace impact in the areas of innovation and socio-economic development. While startups demonstrated substantial contributions in terms of new products, services, data skills, and the creation of jobs, there was low interest in data standardisation and long-term legal strategy. It was therefore difficult to justify our expected impact of improved standardisation and interoperability in the context of cross-sector data applications and technology convergence. This finding was revealed through the evaluation of engagement with different DMS services over time, which is discussed in the next section.

### Increasing engagement

A major finding observed across all dimensions of our results was the increase in engagement with services in the second cohort of the programme. The reasons behind this success were revealed by the workshops that were conducted with DMS partners at different stages of the programme.

In its first year, DMS had already established a quick and agile selection process that attracted high numbers of applications that met the programme KPIs. Project partners contributed to this success by leveraging their local networks to promote the project in wider events (e.g., ’ The Next Web’ [TNW] conference). However, during the first Interim workshop, a number of challenges were identified in regards to the startup selection and onboarding procedure.

One of the most challenged criteria in the applications of the first cohort was related to the management of diversity and inclusivity by each startup. It was suggested that this question discouraged applications from companies who have not yet achieved gender equality. For this reason, the question was rephrased in the next intake to accommodate social and environmental responsibility more broadly. The application form was also extended by asking startups to include a pitch deck and to provide more open-ended responses about the needs and vision of their company. These adjustments helped to ensure that the selected companies in the second DMS cohort were better aligned with the services offered by the programme.

Another challenge identified during the first Interim workshop was a lack of information communicated to startups about the services available at DMS. Moreover, partners reported that startups requested additional personalised support as a way to improve the programme. In response to these limitations that were identified during the first cohort, the programme management and promotion team provided better communication during the second open call about the value proposition of DMS and the services on offer. Moreover, the onboarding process was updated to include a needs assessment and personal calls that helped to build rapport with individual startups. This was accompanied by additional personalised mentoring for cohort two.

Together, the above changes helped to increase engagement across all DMS services between cohort one and cohort two. The magnitude of this impact was further revealed by the quantitative monitoring data that were gathered in each service category across successive cohorts of the programme. By generating specific insights into engagement metrics and the deeper reasons behind them, the mixed-methods monitoring approach enabled DMS to respond and multiply its impact on startups within one iteration, and to demonstrate this impact to the funding body.

### Methodological insights

The specific methods used in our monitoring approach included surveys to startups, programme monitoring metrics and workshops. When used on their own, each of these methods has certain limitations. For instance, the Impact Survey suffered from low response rates among startups, while workshops with partners were limited in representing the subjective viewpoints of the people involved in running the programme. Monitoring methods such as webinar statistics and impact surveys are also likely to have represented different audiences. While any team member could participate and rate webinars, the end-of-year Impact Survey was mostly completed by a single company representative, who may not necessarily be aware of the skills and competencies acquired by all members of personnel who participated in the programme. By triangulating the various pieces of information available to us, we were able to create the in-depth and critical assessment necessary to evaluate and improve the programme. The generalised mixed-methods approach used here, as outlined in the Methods section, can be replicated by other similar initiatives using resources and practices that are already likely to be a part of their monitoring approach.

In addition to methodological triangulation, our findings were classified into four dimensions of impact which made it possible to identify specific areas for improvement as the programme moves into into its next year. The observed increase in engagement across the four service dimensions was not equal, with some areas demonstrating significantly higher engagement than others. In particular, services related to the market and fundraising showed fivefold and threefold increases respectively. Engagement with innovation services had also increased, but with a lower magnitude compared to the other dimensions, suggesting that this is the area that would benefit most from further improvement.

Although a large volume of new products and services was generated by startups in the second cohort, programme monitoring and workshops with partners indicated that there was low interest in training related to long-term innovation strategy and standardisation. Other data competencies such as GDPR and data skills received more interest. The low appeal of standards training complements a separate observation made by programme partners during workshops, where they felt that the focus on *data* in the Data Market Services accelerator is not something that we are sufficiently promoting and showcasing. This was reflected in the needs presented by startups entering the programme, who were most interested in general business competencies such as international mobility, promotion and fundraising.

Possible ways of resolving this gap in the future could involve adjustments to the startup selection procedure to prioritise those that have a stronger motivation to adopt data standards. Additionally, it will be important to develop a more compelling value proposition that communicates the benefits of standardisation to startups who may not yet be aware of opportunities for building data value chains. To support active engagement in this area, we can consider another current limitation related to the lack of communication between participants of the programme. Both issues could be solved by establishing “communities of practice” around data innovation and the adoption of standards.

As discussed in the background literature, standardisation can be an arduous and costly process for businesses. The challenges faced by DMS are likely to be shared by other similar programmes that specialise in data. We hope that this paper will start a discussion about the best ways to monitor and foster the impact of data innovation programmes in Europe.

## Best practice and recommendations

In the beginning of this article, we highlighted the diversity of innovation programmes that have proliferated in the European economy. Due to the variety of funding models and specific objectives, their impact cannot be measured according to the same criteria. While our specific findings may be relevant to other publicly funded data initiatives, what we wish to share in this article is the generalised methodology we have used to derive actionable insights. This methodology can be transferred across a variety of contexts and can be used to self-assess impact even if no other similar programmes are available for comparison. Our recommendations are as follows:

- **Use a variety of methods to measure impact.** Diverse monitoring approaches help to buffer against the limitations of each method. Specifically, there can be limitations of sample size (e.g., low survey response rates), depth of data (quantitative vs. qualitative), and subjective bias (service providers vs. recipients). We suggest that a combination of methods such as surveys, quantitative monitoring tools, and workshops can be synthesised to accommodate the perspectives of service recipients as well as providers. Additionally, different methods can be reconciled to capture individual as well as collective outcomes (in terms of the team members, companies, and ecosystems served by the programme).
- **Measure impact along multiple dimensions.** By classifying monitoring activities into multiple impact areas (e.g., market, funding, innovation, and socio-economic), it is possible to draw comparisons and identify areas that are most in need of improvement, so that resources can be targeted efficiently towards those services. For data programmes in particular, we recommend that special attention is required to assess the role of standardisation and legal strategy in fostering innovation through data value chains.
- **Monitor changes in impact over time.** It is expected that the impact of a programme will increase as it assimilates the learning derived from successive iterations of service delivery. The magnitude of change can serve as an indicator of the agility with which the programme is able to respond to dynamic economic circumstances. Metrics derived as part of routine KPI monitoring activities can be compared between cohorts and interpreted through discussions with programme partners who were involved in service delivery. This can help to identify the actions and strategies that contributed to particular outcomes. In the case of DMS, we learnt that a substantial increase in engagement with services has been achieved, which could be traced back to the self-assessment methodology. Through workshops with partners and surveys completed by startups, DMS was able to identify the precise changes required to improve the selection and onboarding procedures for startups, as well as the services offered to them, such that the companies in the next cohort could benefit maximally from the programme.

In conclusion, our article has discussed some of the challenges of assessing the impact of open innovation programmes related to data and AI. We have proposed a generalised methodological framework that combines multiple monitoring tools and utilises the direct experiences of stakeholders to create meaningful evaluations and actionable recommendations that can improve the social and economic impact such programmes.

## Data and software availability

### Underlying Data

The main contribution of our article is methodological. All data underlying the methodological results are available as part of the article and no additional source data are required.

The underlying survey data cannot be shared openly due to small sample sizes and the respondents’ public affiliation with DMS Accelerator. In order to prevent the possibility of de-anonymisation and to protect commercially sensitive data, access to these data is controlled and classed as confidential as outlined in the ethics statement.

The original routine monitoring and workshop reports cannot be published because they are classified as “Confidential” in the project. A redacted version of the reports can be provided upon request.

To request access to the above survey and workshop data, please email the authors ( maria.1.priestley@kcl.ac.uk and manguiano@zabala.es). Maria Priestley and María Anguiano will evaluate each request and provide access to trusted parties who want to use the data for justified and compatible purposes (e.g. colleagues who want to undertake a comparative research study or a review).

### Extended Data

Zenodo Repository: DMS Accelerator monitoring data ^18^. https://doi.org/10.5281/zenodo.4905499

This project contains the following extended data:

- DMS Impact Survey template
- DMS Needs Analysis Survey template
- DMS Workshop template
- DMS service engagement data

Data are available under the terms of the Creative Commons Attribution 4.0 International license (CC-BY 4.0). 

## Footnotes

^1^ https://opendataincubator.eu/
