# Measuring the impact of publicly funded open innovation programmes: the case of Data Market Services Accelerator

**DOI:** 10.12688/openreseurope.13621.2

**Published:** 2022-04-25

**Authors:** Maria Priestley, Elena Simperl, Cristina Juc, María Anguiano

**Affiliations:** 1 Department of Informatics, King's College London, London, WC2B 4BG, UK; 2 Spherik Accelerator, Bucharest, 400267, Romania; 3 ZABALA Innovation Consulting, Mutilva Alta, Navarra, 31192, Spain

**Keywords:** open innovation, data ecosystems, business accelerators, impact assessment, startups, SMEs

## Abstract

One of the current goals of the European Commission is to stimulate the development and uptake of data and AI technologies in the economy. Substantial funding is being invested in open innovation programmes that help startups and small-medium enterprises (SMEs) to assimilate the latest technical and regulatory trends, and to share their innovations with other organisations. In order to assess the efficacy and impact of such initiatives, their specific social and economic objectives must be taken into consideration. As an example of how this can be done in practice, our paper presents the motivating objectives and methodological approaches that were used to assess the impact of the Data Market Services Accelerator (DMS), an EU-funded initiative for data-centric companies. We evaluated the performance of the programme in terms of its effect on the market, fundraising capabilities of companies, innovation, and socio-economic aspects. In addition to assessing how DMS was able to meet its intended objectives, our examination also underscored current challenges related to specific outcomes that are meaningful to the European Commission, including data standardisation and long-term legal strategy. We conclude the paper with a series of recommendations to support the impact assessment efforts of other similar innovation programmes.

## Executive summary

This paper discusses current challenges related to the measurement of impact in open innovation programmes for datacentric startups and SMEs. Drawing on our experience from the Data Market Services (DMS) Accelerator, we discuss some of the approaches and methods that can help to assess progress towards the specific objectives of publicly funded data innovation programmes in Europe.

Publicly funded data innovation programmes embody a novel form of support for startups and SMEs. While these initiatives share traits in common with traditional business accelerators and incubators in terms of the services offered, public funders strive to fulfil a unique set of objectives. In addition to the financial return generated by individual firms, such initiatives have longer-term goals related to the creation of employment and regional development. Another goal particular to big data initiatives is the creation of data value chains by means of interoperable products and services. Ambitions of this kind require specialist training in legal strategy and standardisation. However, the substantial time and resource investment required from startups to participate in this long-term vision is not always aligned with short-term business goals and profitability. Impact assessments must therefore consider performance at the individual company and ecosystem levels, as well as deriving metrics for ex-ante analysis of wider socio-economic impact.

Based on a review of previous impact assessment approaches, we used a mixed methods framework to evaluate the impact of DMS Accelerator along the following dimensions:

- Market
- Funding
- Innovation
- Socio-economic aspects

Our article provides an initial assessment exercise demonstrating how this framework has been applied in practice at DMS, where methods such as surveys, performance metrics and workshops have been used to obtain monitoring data. Based on insights gathered from startups and programme partners throughout the three-year duration of the programme, we derive methodological recommendations and best practices.

The results of our self-assessment highlighted topical challenges that are relevant to other open innovation programmes that specialise in supporting data-centric startups and SMEs. We found that the DMS Accelerator showed rapid development in dimensions related to the market and fundraising, but was less impactful in some aspects of innovation and socio-economic development. Specifically, there was relatively low interest in standardisation and long-term legal strategy among startups, as well as few opportunities for them to communicate or learn about each other’s inventions. It was therefore difficult to justify our expected impact of improved standardisation and interoperability in the context of cross-sector data applications and technology convergence. We propose that this could be addressed in future by adjusting the startup selection procedure to prioritise companies that have a stronger interest in long-term development, as well as the motivation to improve their collaboration capabilities through standardisation or Intellectual Property Rights (IPR). Additionally, a more compelling value proposition can be developed to communicate the benefits of standardisation and IPR to startups who are not yet aware of opportunities to become part of data value chains. By promoting services related to standards and legal strategy, publicly funded programmes such as DMS Accelerator can help startup founders to understand the value of these practices and incorporate them into their business.

## Introduction

Many societies across the world are undergoing a transformation through technologies affiliated with AI ^1^. This field promises to revolutionise diverse areas of economic activity, including the provision for energy, mobility, food, and healthcare ^2^. These developments rely on data as a critical asset, with data-driven innovation becoming a top priority for technology strategists and policy makers in Europe and globally.

Deriving sustainable benefits from data requires a continuous supply of innovative ideas about how best to solve social, economic and environmental challenges. While this can be a difficult task for mature stakeholders whose business processes are already firmly set, startups and SMEs have the agility to explore, generate, and deploy new radical ideas in the economy ^3^. At the same time, smaller companies face the greatest barriers (in terms of knowledge and resources) to the adoption of digital innovation practices that are advocated by the European Commission, including standardisation and intellectual property rights ^4, 5^. In order to help nascent firms to access the specialist expertise and resources required to overcome these challenges and bring new discoveries to market, corporate and public stakeholders have been active in channeling resources towards them via data-centric business support programmes.

Business accelerators and incubators have been some the most common ways of delivering the support services mentioned above. These programmes started gaining popularity several decades ago, before the emergence of data-centric technologies, as a means of providing nascent firms with advice, services, financing (on occasion), and facilities to help them develop and launch their business ^6, 7^. Subtle differences in the objectives and modes of service delivery have informed the naming conventions of these initiatives. For example, accelerators have traditionally been known to quickly move startups from one stage to the next, while incubators have sought to develop longer-term capabilities that constitute self-sustaining, mature businesses ^6^. Additionally, incubators have tended to target companies from the local community, while accelerators worked with firms at national and international levels ^6^.

Although the labels "accelerator" and "incubator" continue to be used by data-centric programmes, the distinctions between them are less clear-cut in the specific context of data-centric initiatives funded by the EU. An ecosystem of such projects has emerged from a Public Private Partnership (PPP) between the European Commission and the Big Data Value Association (BDVA), with examples including the Data Market Services Accelerator (DMS), European Data Incubator (EDI), EuRopEAn incubator for trusted and secure data value Chains (REACH), MediaFutures, and EUHubs4Data. These support programmes are alike in offering services that address short-term acceleration in *addition to* long-term sustainable innovation goals, which traditionally characterised incubators in the past. Data-centric accelerators and in cubators are also similar in their adoption of an online delivery format, where a diverse range of applicants and service providers are assembled from different geographic regions, which contrasts with traditional incubators that operate locally on site. It is becoming apparent that despite their self-selected labels, EU-funded accelerators and incubators that specialise in the data economy have a convergence of traits that transcend previous notions of these labels. We therefore consider it constructive to refer to them as *open innovation* programmes, a higher-level concept that accommodates their differences and decentralised stakeholder configurations. 

“Open innovation” is a paradigm that describes the “purposive inflows and outflows of knowledge [between organisations] to accelerate internal innovation and expand the markets for external use of innovation” ^8^. Instead of keeping the internal development of products and services within a single firm, open innovation draws upon the collaborative capacity and collective expertise of a wider variety of participants. In addition to multi-stakeholder incubation and acceleration programmes such as those mentioned above, contemporary examples of open innovation include innovation contests, knowledge transfer partnerships, corporate business labs and hubs, and science parks ^9^.

Applied to the areas of data and AI, open innovation programmes in Europe encourage the transfer of datasets, services, and ideas between parties in multiple ways. On one level, knowledge and resources are exchanged between the collaborative arrangement of public and private stakeholders who develop joint training, mentoring and networking curricula. For example, standardisation bodies and law firms contribute guidance on data compliance, interoperability and intellectual property issues, while accelerators support incoming firms in developing their business strategy and investment plans, and dissemination managers assist their marketing strategy. At another level, these consortia provide guidance to participating startups and SMEs on how to develop their own infrastructures, business models and competencies to reuse data and integrate data products with external organisations, helping them to become agents of open innovation in their own right ^3^. While these efforts, and open innovation more broadly, benefit a variety of stakeholders (e.g. large industry, universities and other research institutions), our article focuses on open innovation programmes targeted specifically towards startups and SMEs as priority stakeholders identified by the European Commission. Given that the funds available for these interventions are a limited resource, effective monitoring and allocation of funds requires programme managers and funders to be able to measure the specific socio-economic benefits achieved by each programme. 

Our article explores the question of how impact can be assessed in open innovation programmes that support datacentric startups and SMEs. Motivated by our work at the DMS Accelerator, we review previous approaches to impact assessment, their position in the wider economic context, and what distinguishes data and AI programmes from other types of business support initiatives in the European tech scene. We then present the methodology which was developed and adopted at DMS to monitor the performance and subsequent impact of our services. This paper aims to share our learning with the wider community, discuss best practices, and provide recommendations for policy makers and managers of other similar initiatives.

### DMS and its mandate as part of the BDVA ecosystem

DMS Accelerator is one of the open innovation programmes funded by the European Commission to address the major hurdling blocks faced by startups and SMEs specialising in data. Selected companies are invited to draw from a service catalogue that helps them with fundraising, acceleration, standards, legal, promotion, and data skills. These services are delivered by a consortium of 10 organisations that include European accelerators, dissemination managers, standardisation bodies and universities. Overall, the programme services 150 startups and SMEs over the span of three cohorts, one per year. Unlike other programmes that offer seed funding, the DMS offer consists entirely of services that are delivered free of charge.

DMS is part of a broader ecosystem of projects associated with the Big Data Value Association (BDVA). Since 2014, the BDVA has partnered with the European Commission to support the development of jobs, products, and services around Big Data in Europe. In addition to regional development, this partnership intends to support data markets and data value chains. Both of these rely on standardisation as a mechanism to support interoperability between existing data sources and services, providing a foundation for future recombinant innovations that address previously unforeseen uses ^5^. For instance, standardised data formats reduce the time and effort required by an external user to incorporate the resource into their own solution, and associated services (e.g., Application Programming Interfaces [APIs]) make it possible for others to benefit from the solution without having to reinvent the same service. Together, compliance with common data standards adds value to the data ecosystem by supporting long-term innovation and interoperability. This is necessary formeeting the European Commission’s desire to achieve technology convergence and new cross-sector applications (e.g., in relation to Cloud, Internet of Things [IoT], and Privacy Preserving Technologies).

One of the difficulties in achieving the above goals is that the economic benefits of standardisation may not be immediate for individual startups. Researchers have highlighted that AI companies face a trade-off between pursuing standardisation for the benefit of compatibility with other platforms versus a better fit and flexibility within their business ^10, 11^. Taking the time to learn and implement best practices in relation to data management requires organisations to endure short-term costs, which may be felt most acutely by startups and SMEs ^4^. The full impact of programmes such as DMS must therefore take into account not only the financial success generated by firms who participate in these efforts, but also their actualised and potential impact on the wider entrepreneurial ecosystem over the long-term. Outcomes such as the creation of jobs, data skills, collaborations, products, and reusable data sources lead to economic benefits for other stakeholders that can be monetised at future points in time.

Another aspect of interoperability relates to the creation of common data spaces and common practices for sharing data between different stakeholders. A vision of pan-European data sharing spaces has been supported by the BDVA as a private counterpart of BDV PPP ^12^. Their role is to guide public and private investment towards fair, secure, and legal governance frameworks such that large-scale data can be connected and valorised. In order to exploit the analytic and economic value of distributed data assets within the bounds of the EU regulatory landscape, small businesses are supported in their compliance with the General Data Protection Regulation (GDPR) and adoption of Intellectual Property Rights (IPR). While some programmes, such as DMS, deliver this support entirely through training and mentoring services, other programmes embed practical experimentation and collaboration opportunities with external stakeholders. For example, initiatives such as DataPitch and the European Data Incubator (EDI) entailed ’challenges’ where startups were matched with corporate data providers to develop new data-driven business models and solutions. Endeavours of this kind have culminated in practical learning and recommendations, with examples including the DataPitch Data Sharing Toolkit and the Legal and Privacy Toolkit, which aim to support subsequent data collaborations and build trust in industries affiliated with data and AI.

### Different funding objectives require different impact metrics

There are various kinds of initiatives whose impact assessment approaches can be drawn upon and compared to identify relevant impact metrics for programmes such as DMS. While open innovation programmes share many characteristics in common with traditional business incubators and accelerators, they also cater to specific aspects of the data economy and collective objectives that are more aligned with social innovation programmes. Additionally, the focus on data-driven innovation at DMS resonates with contests and datathons that have acquired a unique set of evaluation metrics. We discuss these approaches below and identify a collection of pertinent criteria to inform the design of our impact assessment methodology.

The similarity of our data-centric open innovation programmes to traditional business incubators and accelerators offers an opportunity to explore how the impact of such programmes has been measured in the past. The classification of different programmes and evaluation of their impact is an ongoing challenge that has been identified in the literature ^13, 14^. Programmes differ in their business models, sources of funding, and services offered. Accordingly, numerous authors have highlighted the need for impact measures to take into account their varying objectives. Clarysse and Van Hove ^15^ identified three emerging archetypes in European accelerators: ecosystem builders (publicly funded), investors (privately funded), and matchmakers (hybrid funding). The distinction between them is summarised in Table 1.

**Table 1.** Key archetypes in accelerators. Adapted from Clarysse and Van Hove ^15^.

Archetype:	Private funding Investor-led	Hybrid funding Matchmaker	Public funding Ecosystem
Strategic focus:	Select attractive investment propositions and turn early–stage projects into profitable businesses.	Typically non-profit orientation. Corporates match startups to their own customers or stakeholders.	Stimulate startup activity within specific regions or technological domains.
Programme package:	Mentorship from serial entrepreneurs and business angels; often sector specific.	Coaching and mentorship from internal experts; especially helping startups to navigate corporate customers.	Mentorship from serial entrepreneurs and business developers; most developed curriculum.
Funding of startups:	Seed funding offered in exchange for equity.	Typically no seed funding.	Various funding structures and revenue models.
Selection:	Favour ventures in later stages with a proven track record.	Favour ventures in later stages with a proven track record.	Favour ventures in very early stages.
Impact assessment:	Revenue	No hard KPIs	Employment

Sources of funding introduce complexity into the identification of assessment criteria for programmes such as DMS. Traditionally, accelerators were funded by investment from business angels, venture capital funds, or corporate venture capital with the intention of capitalising on profitable startups ^15^. The return on investment was the main business model that catalysed the growth of such accelerators, and so impact was measured by the return on investment from startups. The working capital of more recent programmes has relied on shareholders such as investors, corporates and public authorities whose missions are less concerned with short-term financial gain. Instead, these programmes strive to create economic benefits on the wider entrepreneurial ecosystem and in accordance with the sustainable development goals (SDGs).

Publicly funded innovation programmes related to data and AI typically fit across the matchmaking and ecosystem building archetypes presented in Table 1. Examples of past programmes include ODINE, DataPitch, STARTS, and Future Internet (e.g., FINODEX). Current programmes include DMS, REACH, Media Futures, and EUHubs4Data.

Previous systematic studies into the performance of different accelerator models have relied on metrics such as the funding raised and valuation attained by the companies, revealing that graduates of publicly sponsored programs tend to raise significantly lower sums of capital post-accelerator ^16^. Clarysse and Van Hove ^15^ highlight that accelerators financed under the objectives of regional development and employment struggle to be profitable in the short or even medium term. These programmes’ selection criteria and success in meeting socio-economic objectives are not aligned to the creation of profit.

While previous publicly-funded programmes have nonetheless monitored firm-level financial performance as part of their impact assessment, many have also traced the impact on employment, society, and the environment. This has been particularly true of social innovation programmes such as those from the past EU call for “Collective Awareness Platforms for Sustainability and Social Innovation” (CAPS). Within this call, the Impact Assessment for Social Innovation (IA4SI) project defined a mixed methods framework encompassing social, economic, political, and environmental impacts ^17^. Although this example offers a comprehensive methodological structure, it is useful to consider other frameworks that can better accommodate the focal intention of DMS in terms of technological and data-driven entrepreneurship.

Initiatives that have a stronger focus on data innovation offer insight into some of the additional metrics that have been useful in the past. In particular, hackathons and short innovation contests hosted by platforms such as InnoCentive, Top-Coder, and Kaggle provide well-defined challenges where data scientists, researchers, and developers compete to solve complex problems presented by industry or public stakeholders. Such experiments have contributed to the procurement of novel solutions in fields such as health, criminology, and search technology. The impact of innovation contests has been assessed by frameworks such as the ICAPT (Innovation Contests as an Alternative Procurement Tool), which compares the cost of solutions procured through contests relative to the estimated cost of developing an equivalent solution by traditional methods ^18^. This framework also evaluates qualitative benefits such as project awareness and best practices for management. It has been found that contests are cheaper ^19^, and that their collaborative format enables solutions to be discovered much quicker and through a more diverse range of participants ^20^. These past findings imply the utility of impact metrics that assess the cost, speed, and diversity of innovation contests and research experimentation.

The discussion above drew on a range of innovation initiatives whose impact assessment criteria can be relevant to programmes such as DMS. However, we are faced with the challenge of evaluating a programme that sits at the intersection of financial, social, and technical objectives. The desire to support the profitability of individual firms is combined here with a vision for sustainable long-term development and technological innovation, requiring a unified impact assessment framework. There are a number of past and current programmes that tackled similar transversal objectives in their impact assessments:

- **Open Data Incubator Europe (ODINE)** ^1^ was a programme for open data entrepreneurs that reported impact in terms of the number of incubated ideas, return on investment, engagement, jobs, and geographic representation.
- **Data Pitch** matched data providers with startups who worked to address open innovation challenges. Their impact assessment reported on the number of data-driven businesses established, cross-sector and cross-border collaborations, financial impact, and the creation of big-data use cases that drive investment ^21^. Following its completion, the programme organisers developed additional resources that could be used by other organisations. For example, the Data Sharing Toolkit helps organisations to generate value by allowing third parties specifically permissioned access to private datasets.
- **Science, Technology and the Arts (STARTS)** was a residential innovation programme designed to increase the impact of artists in high-tech scientific environments ^22^. They committed to deliver a certain number of residencies, a global methodology and tools to promote collaborative work, as well as knowledge to evaluate success factors in future initiatives.

- **Future Internet (FI)** encompassed a number of accelerators working in Internet-enabled innovation ^23^. The project developed analytic methods and tools to perform an ex-ante socio-economic impact analysis. Their frame-work contained several assessment areas and KPIs including:
**Market** - customers, revenue, and geographies (footprint in EU economy)
**Socio-economic** - social, scientific, and macro-economic consequences (e.g., wider perception of AI)
**Innovation** - types of technology solution, intellectual property (IPR)
**Funding** - funding requested and quality of financial plan

- **DataBench** is currently creating a benchmarking process for organisations that develop Big Data Technologies (BDT) ^24^. The framework measures technology development activity against parameters of high business relevance, seeking to demonstrate industrial significance and return on investment.

### Methodological implications

What unites the programmes listed in the previous section is that their impact assessment approaches drew on a variety of metrics to capture financial, social, and technological outcomes. Additionally, they presented nuanced methodological considerations in terms of benchmarking and assessing qualitative impacts. In the following paragraphs we discuss what the quantitative and qualitative impacts commonly entail, and suggest ways of synthesising these strengths into a generalisable impact assessment approach.

When it comes to quantitative metrics, a number of earlier programmes have been able to conduct rigorous evaluations of impact through counterfactual analysis. For example, Data Pitch compared their outcomes with what would have been achieved by startups in the absence of the programme ^21^. This kind of analysis is conceptually similar to benchmarking approaches such as Data Bench, where the impact assessment is based on relative comparisons. However, it is important to consider that not every programme will have the resources to benefit from counterfactual analysis or benchmarking tools. In the present case, we propose the comparison of measurements across multiple cohorts of the same programme as a simpler way to assess impact in terms of temporal improvement and agility in the specific attributes relevant to that programme.

When evaluating qualitative impacts, previous data innovation programmes were alike in reporting on their methodological contributions and learning. Publicly funded accelerators are characterised by high quality self-assessment and transparent reporting of outcomes, often sharing expertise that has an impact on the wider BDVA ecosystem. Due to the limited duration of publicly-funded programmes (DMS lasts three years) it is not usually possible to measure the actualised impacts and value chains that accrue over the longer term. However, resources that have been released into the ecosystem for future use are one of the ways of ensuring lasting impact. We therefore propose viewing methodological contributions as one of the manifestations of socio-economic impact. In the case of the DMS Accelerator, one of the defining qualities of the programme is that it attracts startups based purely on its services, without offering seed funding. The methods that were developed for selecting and onboarding startups who are motivated and engaged by this service proposition could therefore be an impactful resource for other accelerators whose funding model is similar to that of DMS.

In addition to revealing impact assessment approaches that are held in common by multiple programmes, our overview also highlighted challenges for developing a unified impact assessment methodology. Some past programmes such as Data Pitch were structured in such a way that startups were systematically matched with data providers, making it possible to demonstrate well-defined data experiments and collaborations at the broader socio-technical level. However, because this indicator relied on the specific format of the programme, it would be difficult to replicate the same assessment in another programme such as DMS. One of our intentions in this article is to identify a flexible and generalisable framework, which is applicable to DMS but not endogenous to it.

We suggest that the main impact categories defined previously by the FI-Impact project can be used as the basis for such a framework. Specifically, the categories of 1) Market, 2) Funding, 3) Innovation, and 4) Socio-economic aspects provide a comprehensive yet generalisable representation of the main areas of impact applicable across publicly funded data innovation programmes. In addition to assessing impact on the entrepreneurial ecosystem in terms of sales and investment, the dimensions also accommodate technical advances and qualitative socio-economic impacts. For the purposes of flexibility, the specific metrics within each impact category can be defined in accordance with the monitoring opportunities of particular programmes. A variety of qualitative and quantitative methods can be combined to assess each impact dimension during successive years of the programme. In the remainder of this article, we demonstrate how the four-dimensional impact framework has been implemented in practice at DMS Accelerator, and the results gained from our experience.

## Methods

The DMS programme is unique in its focus on service provision, rather than pre-defined data challenges or collaborations that have characterised other similar programmes in the past. Our methodological framework therefore has an emphasis on the evaluation of services (in terms of engagement and satisfaction), while at the same time incorporating financial successes of firms, new products, collaborations, and public awareness metrics. DMS services are already classified into categories (Acceleration, Promotion, Fundraising, Standards and Legal, and Data Skills) that lend themselves to the FI-Impact dimensions (Market, Funding, and Innovation). Moreover, the expected impacts of the DMS programme that are outlined in the grant agreement also map onto all four dimensions, including Socio-Economic impact. These expected impacts are presented in Table 2 (first column).

**Table 2.** Impact assessment methodology proposed for DMS.

Dimension Followed by expected and additional impacts as specified in the DMS grant agreement	Individual company metrics	Collective metrics / KPIs
**Market** DMS expected impact: • At least 50 clients (e.g., start-ups, SMEs) served annually in partner finding, matchmaking, venture capital raising, training, and coaching • Reach 2,000 companies out of which 150 will be serviced • Increase their sales capacity by 15%	• Sales capacity • Revenue • New clients	• Number of contacted and recruited companies • Companies at different stages of development • Representation of different industry sectors and countries • Engagement in acceleration webinars
**Funding** DMS additional impact: • New rounds of private capital reaching 5m euros • Additional public funds reaching 1m • Capacity for future investment and partnerships offered by the consortium and Advisory Board of investors	• Additional funding gained	• Engagement in fundraising webinars • Meetings with investors
**Innovation** DMS expected impact: • Offer training on standards and legal issues to 150 companies • Improved standardisation and interoperability especially in the context of cross-sector applications and technology convergence (data, Cloud, IoT, connectivity)	• New products, datasets, services • Patents	• Engagement in standards & legal webinars • Engagement in data skills courses • Mentoring sessions in standards & legal
**Socio-economic** DMS expected impact: • Success stories as a result of services offered • Dissemination and exposure of success stories DMS additional impact: • At least 200 new jobs requiring data skills will be created in the portfolio of companies	• Jobs created • Gender composition of teams • Collaborations • Success stories	• Social media followers • Website visitors • Audience at events • Self-organised events • Methodology shared with other programmes
Method of assessment:	Surveys supplemented with desk research	Programme monitoring metrics and workshops with programme partners

The four-dimensional impact criteria help to address the tension between the short-term and long-term economic impacts of data innovation programmes. At DMS, this is achieved through metrics that monitor impact at two levels of analysis: 1) the success of companies who complete the programme, and 2) impact on the collective ecosystem (the entrepreneurial environment as well as on other open innovation programmes). We evaluate these outcomes using a mixed-methods approach. Quantitative metrics are derived from programme monitoring activities and close-ended survey questions completed by startups when they leave the project. Qualitative approaches were additionally used to gain a more nuanced understanding of these metrics. For example, open-ended survey questions were used to assess the ways, if any, that participation in DMS contributed to the successes of startups in specific dimensions. We also conducted workshops with key partners from DMS to examine the collective impact of the programme and observations that were not covered by routinely monitored KPIs.

To summarise, the results presented in this article are based on programme monitoring metrics, surveys completed by startups, and workshops with DMS partners. Ethical considerations surrounding these data sources are discussed below, followed by descriptions of how each type of data was gathered.

### Ethics statement

Our investigation reuses data that were collected by the DMS Accelerator as part of its routine monitoring activities. The data are fully anonymised and we have obtained verbal consent from the DMS partners for our secondary use of the data. When collecting information, DMS informs participating companies that the gathered data may be used for research and reporting purposes. Additional consent to openly publish the data was not obtained due to the inclusion of commercially sensitive information that needs to remain private as outlined in the data availability statement. As our study is low risk and does not use personal data, approval from the King’s College London ethics committee was not required. Our study was done in consultation with the Information Compliance team at King’s College London.

**Routine monitoring metrics** were sourced from records kept throughout the programme, where available. This includes engagement and dissemination KPIs reported by DMS in its periodic reports from the years 2020 and 2021, as well as webinar and course engagement statistics. Our analysis includes all data that fall within the scope of the expected and additional impacts presented in the DMS grant agreement, as listed in the first column of Table 2. For the purposes of brevity, we have excluded information relating to specific events and webinars conducted by DMS. Instead, the metrics are reported here in aggregate form to reflect the average attendance and satisfaction in each service category.

**Survey responses** were gathered using an ’Impact Survey’ delivered to all three cohorts of graduates from the DMS programme. The first two cohorts were surveyed twice, once upon leaving the programme and then one year later using a follow-up survey. Blank templates of the survey are available as described in the *Data Availability* statement ^25^. The survey contained a selection of multiple-choice, closed-ended, and open-ended questions related to topics that are summarised in the middle column of Table 2. The closed-ended questions were compulsory, while the open-ended questions that followed them were optional. Participants who did not provide written answers for a particular question were not included in the results reported for that question. The questions were slightly different in each DMS cohort in order to account for differences in the amount of time that had elapsed since they completed the programme. The coordinator of DMS Accelerator distributed these surveys to each cohort using an email that invited the companies to submit their responses in a Google Form. Reminder emails were also sent, and DMS mentors encouraged companies to respond where possible. The response rate was 26% (13 out of 50) from cohorts one and three, and 42% (21 out of 50) from cohort two. Due to the temporal setup of the impact survey, the responses of cohorts one and two reflect outcomes that were achieved within 11 months after the startups left the programme, whereas the responses of Cohort three reflect their immediate outcomes.

**Workshop outcomes** are based on two two-hour workshops conducted with DMS partners. One of these was the Interim workshop which took place on 10th December 2019, in Leipzig, Germany. This was attended by 13 participants from nine partner organisations who had already been invited to be part of the DMS Consortium meeting on the same date. The workshop used a structure that alternated between small group discussions and plenary discussions, forming three 30 minute blocks where participants discussed topics related to startup selection, service provision, and acceleration. Participants were provided with prompts inviting them to comment on what went well, what could have gone better, and what could be done to improve the programme. The results of this workshop were summarised in a confidential Workshop Report deliverable at DMS. Subsequent actions were also taken by the relevant DMS partners to modify the programme’s startup selection and acceleration process in accordance with the recommendations raised by the consortium. In the second year, DMS organised a White Paper workshop on 12th November 2020. 11 participants attended from seven DMS partner organisations, who were invited to participate in a Microsoft Teams meeting. As with the previous workshop, this setup alternated between small group discussions in breakout rooms and joint plenary discussions. There were three 30 minute blocks where participants discussed the most and least successful aspects of the programme, how impact should be defined and assessed, and ways to make the programme more successful in future. The outcomes of this workshop informed the design of the DMS Impact Survey and the content of this paper. A summary of the structure of both workshops is available in the *Extended Data* repository ^25^.

## Results

Our findings are grouped into sections according to the four dimensions of our impact assessment framework: Market, Funding, Innovation, and Socio-economic aspects. The results for each dimension are a triangulation of the methods described above, in the form of monitoring activities, Impact Surveys completed by startups and workshop participation from programme partners.

### Market

In the dimension of the market, our methodology sought to measure the footprint created by the DMS programme on the EU economy. As part of this, we examined the diversity of companies serviced by the project, their engagement with entrepreneurial training and their own capacity to serve new clients. Our assessment of market impact is summarised in Table 3.

**Table 3.** Assessment of market impact.

	Cohort One	Cohort Two	Cohort Three
**Impact Survey** ** answers from ** **startups**	(13 responses in total, 7 surveyed immediately and 6 one year later using a follow-up survey) • Sales capacity (assessed only in follow-up survey) – 3 companies reported an increase in sales capacity. • Revenue – 6 companies reported an increase in revenue. • New clients – 7 companies gained potential new clients. Open-ended answers indicated that the effect of DMS on market impact was achieved by means of improved public image (e.g., through promotional videos and improved negotiation capacity).	(21 responses in total, 15 surveyed immediately and 6 one year later) • Sales capacity – 9 companies reported an increase in sales capacity. • Revenue (assessed only in follow-up survey) – 6 companies reported an increase in revenue. • New clients – 3 companies gained new clients as a result of joining DMS. Open-ended answers mentioned improved company profile and marketing, increased reach and partnerships, improvement in knowledge, and selling proposition.	(13 responses, surveyed immediately) • Sales capacity – 6 companies reported an increase in sales capacity. • Revenue was not included in the survey, as we did not expect an immediate impact. • New clients – 4 companies gained new clients. Open-ended answers mentioned improved strategy, visibility in the market, better lead generation and training.
**Programme ** **Monitoring**	• Contacted companies: 690 • Company stages: 15 scaling, 30 validating, five establishing • Countries represented in portfolio: 16 • 31 mentoring sessions for 10 startups Participation in webinars: • 10 entrepreneurial webinars, five participants on average • Two promotional webinars, three participants on average	• Contacted companies: 1,172 • Company stages: 15 scaling, 30 validating, five establishing • Countries represented in portfolio: 20 • 63 mentoring sessions for 23 startups Participation in webinars: • 11 entrepreneurial webinars, 24 participants on average, average rating 4.17/5 • Three promotional webinars, 14 participants on average, average rating 4.15/5	• Contacted companies: 1,653 • Company stages: 15 scaling, 30 validating, five establishing • Countries represented in portfolio: 17 • 104 mentoring sessions for 28 startups Participation in webinars: • 14 entrepreneurial webinars, 24 participants on average, average rating 4.15/5 • Three promotional webinars, 9 participants onaverage, average rating 4.27/5
**Workshops with** ** partners**	DMS partners felt that the selection process wassuccessful in attracting a diverse range of companies,representing a strong portion of the EU market. However, the service offering was not communicatedclearly in relation to the needs and business models of startups. Low participation in webinars was flagged as an important challenge to address	Based on feedback after cohort one, DMS improved its communication of services and onboarding process for startups, leading to increased engagement with all training. More personalised mentoring was provided. The market impact of startups also benefited from corporate videos created by DMS.	

**Figure 1.**
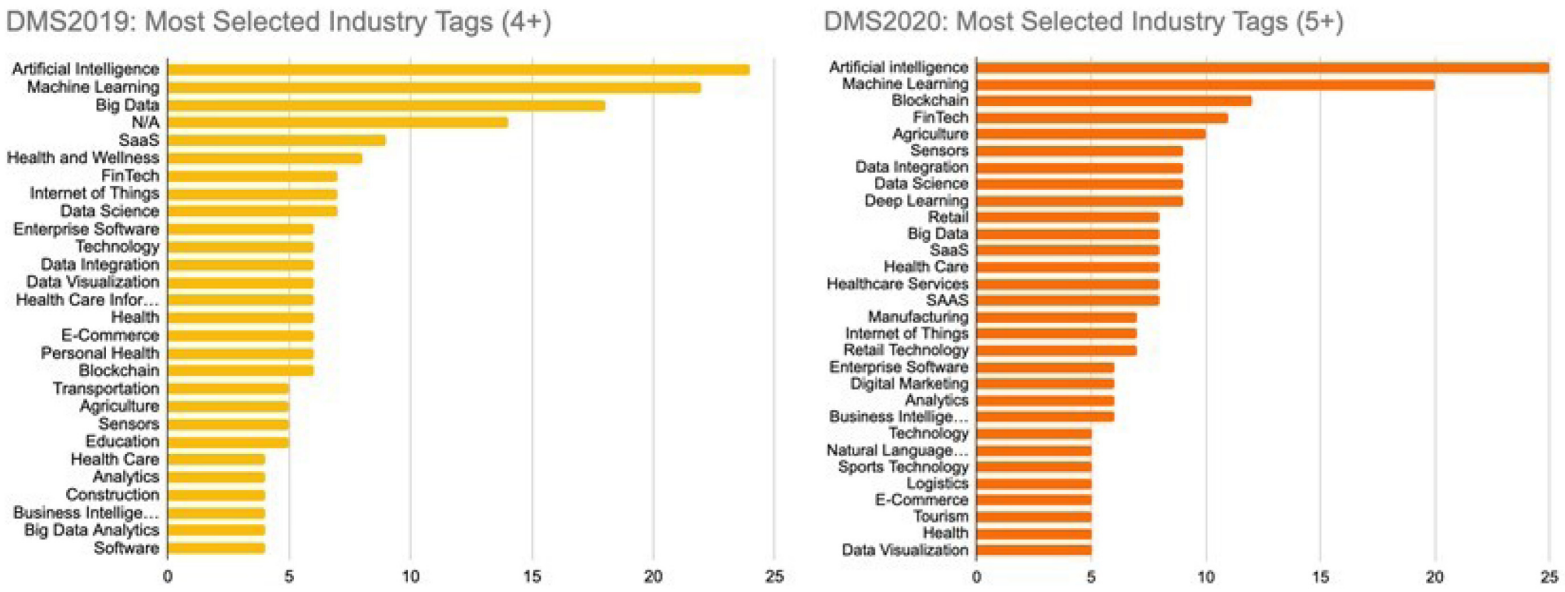
DMS applications in 2019 (left) and 2020 (right) classified by industry tags.

There are a number of ways through which DMS ensures market diversity, and these are part of the routinely monitored KPIs. The first intention is to purposefully accept companies from different stages of development, with 15 scaling, 30 validating, and five establishing companies selected each year. Between the first and subsequent years of the programme, an increase was achieved in the pool of contacted companies, the number of countries represented in the selected portfolio and the representation of AI, ML, and other industry sectors ( Figure 1).

After its first year, programme monitoring and workshop activities highlighted a dramatic increase in the level of engagement with DMS promotional and entrepreneurial services. For example, the number of webinar participants in the Acceleration category increased fivefold from cohort one to cohort two. While it is difficult to assess the extent to which these and other DMS services directly influenced the market success of companies, the results of the Impact Survey from all three cohorts indicate that 18 companies (approximately 45% of those who responded to this question) saw an increase in sales capacity. In the optional question that followed, 10 of these respondents provided a quantitative estimate of their increase, amounting to an average of 36% increased sales capacity per company. A further three respondents mentioned qualitative improvements in general process development, product development and pilot studies that helped to improve their market impact. Besides sales capacity, 14 companies (30% of all respondents) reported gaining new clients, with approximately 3–4 new clients on average among the 12 respondents who provided detail. Open-ended survey answers showed that the training and promotional services offered by DMS helped to improve their companies’ image and reach. Workshops with partners reflected these comments and established that one of the main sources of such impact came from professional videos that were created for the start-ups, who were then able to publish them on their own social media or website.

Together, the above findings show progress towards meeting the expected market impacts of the programme, especially in KPIs related to the diversity of the audience and the number of startups served. The target of increasing startups’ sales capacity by 15% has been more difficult to confirm due to low survey response rates as shown in Table 3. Among those who responded, there appears to be a split where half of the companies saw no increase at all, while others reported a substantial increase. This difference in outcomes is consistent with the results observed in other accelerators, whose overall impact can be traced to a much smaller portion of serviced companies that become highly successful ^16^.

### Funding

In the dimension of funding, we assessed the amount of funding generated by DMS startups since starting the programme. We were also interested in the ways in which DMS services contributed to this success. Our assessment of fundraising impact is summarised in Table 4.

**Table 4.** Assessment of fundraising impact.

	Cohort One	Cohort Two	Cohort Three
**Impact Survey** ** answers from** ** startups**	(13 responses in total, 7 surveyed immediately and 6 one year later using a follow-up survey) • 4 companies gained additional funding, collectively reporting 845K Euros. Open-ended survey answers indicated that startups’ fundraising activities benefited from getting acquainted with DMS accelerators, learning about IP protection, and receiving promotional videos.	(21 responses in total, 15 surveyed immediately and 6 one year later) • 9 companies gained additional funding, collectively reporting 3.6M Euros. Open-ended answers indicated that the startups gained confidence in pitching and acquired a better understanding of funding opportunities. The Pitch Day and TNW feature also initiated some introductions for them.	(13 responses, surveyed immediately) • 2 companies gained additional funding collectively reporting 625K Euros. Open-ended answers indicated that the startups learnt where to seek funding opportunities, how to understand their product, build financial models, and how to make them work.
**Programme ** **Monitoring**	• 40 meetings facilitated with investors • Seven fundraising webinars, five participants on average	• 41 meetings facilitated with investors • Seven fundraising webinars, 18 participants on average, average rating 4.28/5	• 9 meetings facilitated with investors • Seven fundraising webinars, 10 participants on average, average rating 4.10/5
**Workshops with** ** partners**	DMS partners reported that investor pitch decks and matchmaking strategies could be more personalised and tailored to the needs of the beneficiaries.	Fundraising went well this year. A high number of meetings with investors was facilitated and clear startup success stories identified by DMS mentors.	

While the programme has no concrete KPIs related to fundraising among startups, several desired impacts were included in the grant agreement. Specifically, DMS aimed for its startups to raise new rounds of private capital reaching 5m Euros and additional public funds reaching 1m. Capacity for these investments and partnerships was supported by the consortium and Advisory Board of investors.

Impact assessments conducted using surveys suggested that approximately 5M Euros had been raised by 15 companies (32% of respondents). Based on the available data, the proportions of successful DMS graduates seems to be roughly consistent with meta-analyses US accelerators, where 23% of companies were found to be successful in raising significant funds after completing the programme ^16^.

Due to the low survey response rates, the topic of funding was investigated with additional desk research using Crunchbase to trace the total funds achieved by DMS graduates since leaving the programme. This revealed that more than 55M in private funding and more than 1.5M in public funding had been raised in reality. When asked about the ways in which DMS contributed to their fundraising activity, companies that responded to the impact survey commented on their improved confidence in pitching and a better understanding of funding opportunities, as well as their improved image developed through the promotional services of DMS. Alongside this, programme monitoring statistics showed improved engagement with services related to fundraising after the first cohort, where the number of participants in fundraising webinars had more than doubled after the second year of the programme. Through workshops with DMS partners, we also learnt that programme mentors provided personalised guidance and facilitated additional meetings with investors, which contributed to the acquisition of funds and identification of clear success stories among startups. Although investor matchmaking played an important role throughout the entire programme, some data about investor meetings during the third DMS cohort was lost due to a change in the service delivery format and tracking mechanisms.

### Innovation

In the dimension of innovation, our methodology sought to capture novel technology solutions and intellectual property as indicators of impact. This dimension encompassed the role of data regulations, legal strategy, standardisation, and interoperability in supporting data value chains. Our assessment of innovation impact is summarised in Table 5.

**Table 5.** Assessment of innovation impact.

	Cohort One	Cohort Two	Cohort Three
**Impact Survey** ** answers from ** **startups**	(13 responses in total, 7 surveyed immediately and 6 one year later using a follow-up survey) • 6 companies developed new products or services Open-ended answers indicated that startups used DMS feedback to refine their product, improve their GDPR compliance and put data management at the core of their business.	(21 responses in total, 15 surveyed immediately and 6 one year later) • 11 companies developed new products or services Open-ended answers reported that DMS helped startups with GDPR compliance, product development strategy and data science skills.	(13 responses, surveyed immediately) • 5 companies developed new products or services Open-ended answers reported that DMS helped startups with their IPR strategy, innovative ideas andfunding awareness.
**Programme ** **Monitoring**	• Eight mentoring sessions in standards and legal requested by seven startups • Three Data Skills courses, 18 participants average • Seven Standards & Legal webinars, nine participants on average, 3.8/5 average rating	• 10+ mentoring sessions in standards and legal • Three Data Skills courses, 18 participants average • Seven Standards & Legal webinars, nine participants on average, 3.8/5 average rating	• 15+ mentoring sessions in standards and legal • Two Data Skills courses, 23 participants average • Seven Standards & Legal webinars, seven participants on average, 4.7/5 average rating
**Workshops** ** with partners**	New services were proposed in relation to intellectual property rights (IPR) and IP mentoring.	Mentoring sessions on GDPR and IPR received excellent feedback. However, delivery was constrained by limited resources and the scope of these services was not clearly distinguished from consultancy. Low commitment and awareness of standardisation among SMEs made it difficult to engage them with services related to standards.	

DMS fulfilled its expected impact of delivering training on standards and legal issues related to data. Programme monitoring activities showed that engagement with Data Skills courses had tripled in the span of three years. Survey responses from startups also indicated that the portion of startups who developed new products or services had almost doubled in the second year, but declined again in the third cohort, which may be attributed to the shorter length of time covered by the survey. The products included web services, mobile and cloud platforms for various sectors, demonstrating potential alignment with the DMS expected impact of fostering cross-sector applications, and technology convergence. In their written answers, the survey respondents reported benefiting from DMS services related to compliance with GDPR and product development strategy. This feedback was reflected in the comments shared by DMS partners during workshops, where they reported that mentoring sessions on GDPR and IPR received excellent feedback.

In addition to positive feedback, workshops with partners revealed a number of limitations related to the uptake of standards and legal services. While mentoring in GDPR was in high demand, other DMS partners observed a low commitment and awareness of topics related to standardisation and long-term legal strategy (e.g,. intellectual property) among startups. Although services related to standards and legal issues experienced an increase of interest between cohorts, this service category had much lower participation compared to entrepreneurial and fundraising training.

### Socio-economic aspects

In the socio-economic dimension, our methodology sought to measure the direct and indirect consequences of DMS in terms of societal and macro-economic forces. The impacts considered here include employment and the capacity to improve the wider perception of AI among European citizens. Our assessment of socio-economic impact is summarised in Table 6.

**Table 6.** Assessment of socio-economic impact.

	Cohort One	Cohort Two	Cohort Three
**Impact Survey** ** answers from ** **startups**	(13 responses in total, 7 surveyed immediately and 6 one year later using a follow-up survey) • 8 companies reported that their team has grown, collectively generating 22 new jobs. • 4 companies reported a change in the gender composition of their team, with an average increase of 14% in women. • 9 companies pursued new collaborations or partnerships. Open-ended answers reported benefiting from the networking opportunities provided by DMS, but reported needing more personalised support with connecting to investors and collaborators.	(21 responses in total, 15 surveyed immediately and 6 one year later) • 13 companies reported that their team has grown, collectively generating 50 new jobs. • 8 companies reported a change in the gender composition of their team, with an average increase of 0.47% in women (5 teams increased, 2 decreased). • 20 companies pursued new collaborations or partnerships. Open-ended answers reported that DMS helped to reach new audiences and markets, but improvements could be made in the amount of personalised support, connecting to investors and more interaction between startups.	(13 responses, surveyed immediately) • 5 companies reported that their team has grown, collectively generating 6 new jobs (9 jobs created, 3 lost). • 2 companies reported a change in the gender composition of their team, with an increase of 20% in women reported by one of the companies. • 11 companies pursued new collaborations or partnerships. Open-ended answers reported that DMS helped to represent and build a community around their startup, as well as helping them to develop their workforce.
**Programme** ** Monitoring**	• Social media followers: 879 • Website visitors per month: 400+ • Audience at events: 1,600 (Pixels Camp) & 15,492 (TNW) • Self-organised events: 11	• Social media followers (cumulative): 2,215 • Website visitors per month: 2,000+ • Audience at events: 13,240 (at TNW, Pixels Camp was cancelled due to COVID-19) • Self-organised events: 5 Methodology shared with other programmes: • Form for engagement with companies • Impact assessment approach (this paper)	• Social media followers (cumulative): 2,571 • Website visitors per month: 3,000+ • Audience at events: 7,500 (at TNW, Pixels Camp was cancelled due to COVID-19) • Self-organised events: 14 Methodology shared with other programmes: • Form for engagement with companies • Assessment of startup needs • Selection practices of data- centric programmes
**Workshops** ** with partners**	Mentoring sessions, bootcamps and events provided opportunities for startups to interact directly with the beneficiaries of the programme, and this was positively appreciated on both ends.	Promotional campaign of the programme was good, but few opportunities were available for communication between startups. Visibility of the project across the European ecosystem could also be improved.	

Responses to the Impact Survey showed that 26 companies (55% of the respondents) had created new jobs within the duration of the programme or shortly after, collectively reporting 78 new jobs and an average of 2–3 new team members per company. The position types were specified for 43 of the new roles, with approximately 24 roles (52% of the reported) being in technical and data-related fields, 12 roles (26%) in business development, 4 roles (10%) in marketing, and the remainder related to finance, sales and HR. While these observations contribute towards the DMS desired goal of creating 200 new jobs requiring data skills, the magnitude of progress towards that number is difficult to assess due to the low survey response rates from startups after completing the programme.

DMS monitors additional social impacts such as the gender composition of startup teams, their collaborations, and published success stories. Impact Surveys showed that, compared to the first cohort, each of these areas demonstrated an improvement in the second and third cohorts. A small portion of the respondents (14 companies, around 30% of respondents) experienced a change in the gender composition of their team, with the representation of women increasing by an average of 14% per company. A significant portion of the respondents (40 companies, 85% of respondents) reported pursuing new collaborations. These startups reported benefiting from the networking opportunities provided by DMS, but they also requested more personalised support in regards to networking with investors and interacting with other DMS startups. The latter point was also highlighted by DMS partners during workshops, where communication opportunities between startups were identified as a weakness in the current service offering.

At the collective level of analysis, programme monitoring statistics indicated a good promotional campaign, presence at events and a large audience. Through feedback acquired from mentors and Impact Surveys, a number of success stories were identified among startups who benefited from the programme. The dissemination of these stories was increased through the promotional reach of the programme.

In addition to the social impact of DMS in terms of public engagement, we were also interested in assessing its position in relation to other similar programmes. Workshops with DMS partners revealed that there was room to increase the programme’s visibility and create closer partnerships with other open innovation programmes in Europe, especially on regional and local levels. In addition to approaching these connections directly, the outputs of the programme could be used to gain exposure. In particular, deliverables such as the startup selection and onboarding procedure ( DMS deliverable D2.3), assessment of startup needs ( D3.5), and whitepapers on impact assessment ( D4.5, this paper) and selection practices (D4.6) are publicly available, such that other similar programmes can benefit from relevant parts of our methodology..

### The reasons for increased engagement

A major finding observed across all dimensions of our results was the increase in engagement with services beginning with the second cohort of the programme. The reasons behind this success were revealed by the workshops that were conducted with DMS partners.

During the Interim workshop following the first DMS cohort, the project partners expressed that the programme had already established a quick and agile selection process that attracted high numbers of applications and met the programme KPIs. The partners contributed to this success by leveraging their local networks to promote the project in wider events (e.g., ’The Next Web’ [TNW] conference), but a number of challenges were identified in regards to the startup selection and onboarding procedure.

One of the most challenged criteria in the applications of the first cohort was related to the management of diversity and inclusivity by each startup. It was suggested that this question discouraged applications from companies who have not yet achieved gender equality. For this reason, the question was rephrased in the next intake to accommodate social and environmental responsibility more broadly. The application form was also extended by asking startups to include a pitch deck and to provide more open-ended responses about the needs and vision of their company. These adjustments helped to ensure that the selected companies in the second and third DMS cohorts were better aligned with the services offered by the programme.

Another challenge identified during the first Interim workshop was a lack of information communicated to startups about the services available at DMS. Moreover, partners reported that startups requested additional personalised support as a way to improve the programme. In response to these limitations, the programme management and promotion team provided better communication during the second and third open calls about the value proposition of DMS and the services on offer. The onboarding process was also updated to include a needs assessment and personal calls that helped to build rapport with individual startups. This was accompanied by additional personalised mentoring during cohorts two and three.

Taken together, the first workshop with DMS partners served the purpose of generating insight into the reasons behind participants’ low engagement with the programme, enabling the programme to respond and multiply its impact on startups in the next two cohorts. The second workshop re-evaluated these amendments to reveal how they contributed to the increased engagement that was observed across all DMS services in the subsequent iterations of the programme.

## Discussion

Building on prior literature and impact assessment methods in previous open innovation programmes, this article presented a general methodological framework and applied it in the context DMS Accelerator. We have demonstrated how dimensions related to the market, fundraising, innovation and socio-economic aspects can be assessed through a combination of qualitative and quantitative indicators coming from surveys, workshops and monitoring data.

Although it was possible to demonstrate the positive impact of DMS in each of our evaluated dimensions, the magnitude of impact was different in each area, and some outcomes were difficult to map onto what was proposed in the DMS grant agreement. We summarise these findings below and present recommendations for other similar programmes.

### The impact created by DMS

In relation to the market, DMS monitoring data showed high diversity in the developmental stages, geographic origins and industry sectors of companies that were serviced by the programme. Evidence of this was observed in the descriptive data accompanying open call applications and the portfolio of selected companies. The companies captured by the programme represented different levels of maturity (45 scaling, 90 validating, and 15 establishing), they came from different geographic regions (28 countries), and represented around 14 industry sectors. Besides descriptive data about the market coverage of programme participants, the programme also provided marketing training and services that enabled participants to independently exert an impact on the market. This was demonstrated by high participation and engagement with entrepreneurial services, as well as the graduating firms’ reported impacts on their sales capacity and client base. Together, these findings give us confidence that DMS has improved the entrepreneurial capacity of selected companies in Europe.

In the dimension of fundraising, DMS was able to demonstrate strong outcomes in the number and size of investments generated by graduating firms. Much of this information came from external platforms such as Crunchbase, as internal surveys conducted by DMS did not have sufficiently high response rates and temporal tracking of the investments achieved by participants. Nonetheless, survey monitoring data showed that at least some of their successes were attributed to the training and guidance that had been acquired by startups through DMS ^25^.

It was more challenging to trace impact in the areas of innovation and socio-economic development. While startups demonstrated substantial contributions in terms of new products, services, data skills, and the creation of jobs, there was low interest in data standardisation and long-term legal strategy. It was therefore difficult to justify the DMS expected impact of "improved standardisation and interoperability in the context of cross-sector data applications and technology convergence". This finding was revealed through the evaluation of engagement with different DMS services over time, which is discussed in the next section.

### Methodological insights

The specific methods used in our monitoring approach included surveys to startups, programme monitoring metrics and workshops. When used on their own, each of these methods has certain limitations. For instance, the Impact Survey suffered from low response rates among startups, while workshops with partners were limited in representing the subjective viewpoints of the people involved in running the programme. Monitoring methods such as webinar statistics and impact surveys are also likely to have represented different audiences. While any team member could participate and rate webinars, the end-of-year Impact Survey was mostly completed by a single company representative, who may not necessarily be aware of the skills and competencies acquired by all members of personnel who participated in the programme. By triangulating the various pieces of information available to us, we were able to create the in-depth and critical assessment necessary to evaluate and improve the impact of DMS. Although the applicability of our mixed-methods approach to other similar initiatives was not tested here, we believe that this methodology can be replicated using resources and practices that are already likely to be a part of other programmes’ monitoring approach.

In addition to methodological triangulation, our findings were classified into four dimensions of impact which made it possible to identify specific areas for improvement. The observed increase in engagement across the four service dimensions was not equal, with some areas demonstrating significantly higher engagement than others. In particular, services related to the market and fundraising showed fivefold and threefold increases respectively. Engagement with innovation services had also increased, but with a slightly lower magnitude compared to the other dimensions, suggesting that this is area could benefit most from further improvement.

Although a large volume of new products and services was generated by startups in the second cohort, programme monitoring and workshops with partners indicated that there was low interest in training related to long-term innovation strategy and standardisation. Other data competencies such as GDPR and data skills received more interest. The low appeal of standards training complements a separate observation made by programme partners during workshops, where they felt that the focus on *data* in the Data Market Services accelerator is not something that we are sufficiently promoting and showcasing. This was reflected in the needs presented by startups entering the programme, who were most interested in general business competencies such as fundraising, promotion, and international mobility ( DMS D3.5).

Possible ways of resolving this gap in the future could involve adjustments to the startup selection procedure to prioritise those that have a stronger motivation to adopt data standards. Additionally, it will be important to develop a more compelling value proposition that communicates the benefits of standardisation and intellectual property to startups who may not yet be aware of opportunities for building data value chains. To support active engagement in this area, we can consider another current limitation related to the lack of communication between participants of the programme. Both issues could be solved by establishing “communities of practice” around data innovation and the adoption of standards.

As discussed in the background literature, standardisation can be an arduous and costly process for businesses. The challenges faced by DMS are likely to be shared by other similar programmes that specialise in data. We hope that this paper will start a discussion about the best ways to monitor and foster the impact of data innovation programmes in Europe.

### Limitations and future work

An important limitation of our study is that it focused on measuring the impact of only one initiative. This presented difficulties in the form of small sample sizes during data collection, as well as limited representation of open innovation programmes more broadly. As noted in the introduction of this article, open innovation occurs not only through business accelerators and incubators, but also via contests, knowledge transfer partnerships, corporate labs and hubs, and science parks. In order to confirm if and how our impact assessment approach is generalisable to other programmes, additional studies would be needed to replicate our conceptual and methodological framework in different settings.

Another limitation of our study relates to theoretical literature. Our study sits in the specific context of data-centric open innovation programmes that are publicly funded. Given that these types of programmes are still somewhat new in relation to the wider open innovation movement, there are not as many scientific publications that cater to the unique characteristics and impact assessment practices of data innovation and data entrepreneurship programmes. Our paper contributed a small step towards this expanding discussion, and we are hopeful that other similar studies are already adding greater scientific rigour to the question of how impact can be measured in data-centric programmes.

## Best practice and recommendations

In the beginning of this article, we highlighted the diversity of innovation programmes that have proliferated in the European data economy. Due to their varied funding models and objectives, their impact cannot be measured according to the same criteria. While our specific findings may be relevant to other publicly funded data initiatives, what we wish to highlight here is the generalised methodology that has helped us to derive actionable insights. We would like to propose the following recommendations to other initiatives similar to DMS who may be seeking to monitor and demonstrate their impact to the funding body:

- **Use a variety of methods to measure impact**. Diverse monitoring approaches help to buffer against the limitations of each method. Specifically, there can be limitations of sample size (e.g., low survey response rates), depth of data (quantitative vs. qualitative), and subjective bias (service providers vs. recipients). We suggest that a combination of methods such as surveys, quantitative monitoring tools, and workshops can be synthesised to accommodate the perspectives of service recipients as well as providers. Additionally, different methods can be reconciled to capture individual as well as collective outcomes (in terms of the team members, companies, and ecosystems served by the programme).
- **Measure impact along multiple dimensions**. By classifying monitoring activities into multiple impact areas (e.g., market, funding, innovation, and socio-economic), it is possible to draw comparisons and identify areas that are most in need of improvement, so that resources can be targeted efficiently towards those services. For data programmes in particular, we recommend that special attention is required to assess the role of standardisation and legal strategy in fostering innovation through data value chains.
- **Monitor changes in impact over time**. It is expected that the impact of a programme will increase as it assimilates the learning derived from successive iterations of service delivery. The magnitude of change can serve as an indicator of the agility with which the programme is able to respond to dynamic economic circumstances. Metrics derived as part of routine KPI monitoring activities can be compared between cohorts and interpreted through discussions with programme partners who were involved in service delivery. This can help to identify the actions and strategies that contributed to particular outcomes. In the case of DMS, we learnt that a substantial increase in engagement with services has been achieved, which could be traced back to the self-assessment methodology. Through workshops with partners and surveys completed by startups, DMS was able to identify the precise changes required to improve the selection and onboarding procedures for startups, as well as the services offered to them, such that the companies in the subsequent cohorts could benefit maximally from the programme.

In conclusion, our article has discussed some of the challenges of assessing the impact of open innovation programmes related to data and AI. We have proposed a generalised methodological framework that combines multiple monitoring tools and utilises the direct experiences of stakeholders to evaluate and develop actionable recommendations that can improve the social and economic impact such programmes.

## Data and software availability

### Underlying data

The main contribution of our article is methodological. All data underlying the methodological results are available as part of the article and no additional source data are required.

The underlying survey data cannot be shared openly due to small sample sizes and the respondents’ public affiliation with DMS Accelerator. In order to prevent the possibility of de-anonymisation and to protect commercially sensitive data, access to these data is controlled and classed as confidential as outlined in the ethics statement.

The original routine monitoring and workshop reports cannot be published because they are classified as “Confidential” in the project. A redacted version of the reports can be provided upon request.

To request access to the above survey and workshop data, please email the authors (maria.1.priestley@kcl.ac.uk and manguiano@zabala.es). Maria Priestley and María Anguiano will evaluate each request and provide access to trusted parties who want to use the data for justified and compatible purposes (e.g. colleagues who want to undertake a comparative research study or a review).

### Extended Data

Zenodo Repository: DMS Accelerator monitoring data ^25^. https://doi.org/10.5281/zenodo.6401138

This project contains the following extended data:

- DMS Impact Survey template
- DMS Needs Analysis Survey template
- DMS Workshop template
- DMS service engagement data

Data are available under the terms of the Creative Commons Attribution 4.0 International license (CC-BY 4.0).

## Footnotes

^1^ https://opendataincubator.eu/
