# Supplementary material for: Measuring the impact of publicly funded open innovation programmes: the case of Data Market Services Accelerator
Source: Open Res Eur. 2022 Jul 28;1:71. Originally published 2021 Jun 18. [Version 3] doi: 10.12688/openreseurope.13621.3 (PMC10445846; doi:10.12688/openreseurope.13621.3)
Supplement: Peer review history

# Peer review: Reviewer response for version 3

**DOI:** 10.21956/openreseurope.16175.r29771

**Published:** 2022-09-07

**Authors:** Gernot Pruschak

**Affiliations:** 1 Business School, Bern University of Applied Sciences, Bern, Switzerland

Dear authors,

 Thank you for the literature additions. They now situate the paper better in the context of Open Innovation. Therefore, I recommend this paper to be approved.

 Kind regards,

 Gernot Pruschak

If applicable, is the statistical analysis and its interpretation appropriate?

No

Is the study design appropriate and is the work technically sound?

Partly

Is the work clearly and accurately presented and does it engage with the current literature?

No

Are the conclusions drawn adequately supported by the results?

No

Are sufficient details of methods and analysis provided to allow replication by others?

Partly

Are all the source data and materials underlying the results available?

Yes

Reviewer Expertise:

Open Innovation, Open Science, Open Data, Quantitative Research, Surveys, Descriptive Statistics, Parametric Statistics

I confirm that I have read this submission and believe that I have an appropriate level of expertise to confirm that it is of an acceptable scientific standard.

# Peer review: Reviewer response for version 2

**DOI:** 10.21956/openreseurope.15814.r29098

**Published:** 2022-05-23

**Authors:** Gernot Pruschak

**Affiliations:** 1 Business School, Bern University of Applied Sciences, Bern, Switzerland

Dear authors,

 Thank you for taking the time and updating the manuscript. The authors put in substantial efforts in revising the manuscript and detailly responded to all my comments. I especially congratulate the authors for increasing the sample size.

 Nevertheless, whereas the authors addressed most of my comments successfully, there still exists room for improvements in two areas: 1) Terminology and 2) Embodiment in the (Scientific) Literature. However, both of these issues can be solved via the same mean:

 The authors claim that data-centric innovation is a relatively new phenomenon, a statement that is definitely true. However, at the same time the authors put in lengthy efforts in elaborating that the focus of their research are not accelerators and incubators but instead open innovation programs. If this is the case, they might also embody their research in the wider open innovation literature. As a case in point, there exists ample literature discussing open data, especially in the public domain (e.g., governments and universities). It would be therefore great if the authors could situate their research more in context with recent developments in the open data literature. This would boost the scientific background of the paper and make it clear to readers why the focus is on open innovation and not on accelerators and incubators.

If applicable, is the statistical analysis and its interpretation appropriate?

No

Is the study design appropriate and is the work technically sound?

Partly

Is the work clearly and accurately presented and does it engage with the current literature?

No

Are the conclusions drawn adequately supported by the results?

No

Are sufficient details of methods and analysis provided to allow replication by others?

Partly

Are all the source data and materials underlying the results available?

Yes

Reviewer Expertise:

Open Innovation, Open Science, Open Data, Quantitative Research, Surveys, Descriptive Statistics, Parametric Statistics

I confirm that I have read this submission and believe that I have an appropriate level of expertise to confirm that it is of an acceptable scientific standard, however I have significant reservations, as outlined above.

# Response

**Published:** 2022-07-27

**Authors:** Maria Priestley

**Affiliations:** 21503, UK

Dear Dr. Pruschak,

 Thank you very much for your additional comments on our revised article. We have now uploaded a new version which incorporates your helpful feedback. The terminology and positioning of our article in the wider scientific literature has been clarified using the following changes:

- The Abstract has been updated to specify that this work builds on earlier open innovation initiatives related to open data.
- A new paragraph has been added to the Introduction section, which now includes a short discussion of the open data movement and references to research in this area.

We would like to thank you once again for your time reviewing our work. We hope that the new version of the article meets your expectations.

 Best wishes,

 Maria

# Peer review: Reviewer response for version 2

**DOI:** 10.21956/openreseurope.15814.r29097

**Published:** 2022-04-25

**Authors:** Francesco Mureddu

**Affiliations:** 1 The Lisbon Council, Brussels, Belgium

I am satisfied with the amendments

If applicable, is the statistical analysis and its interpretation appropriate?

Yes

Is the study design appropriate and is the work technically sound?

Yes

Is the work clearly and accurately presented and does it engage with the current literature?

Yes

Are the conclusions drawn adequately supported by the results?

Yes

Are sufficient details of methods and analysis provided to allow replication by others?

Yes

Are all the source data and materials underlying the results available?

Yes

Reviewer Expertise:

Impact assessment and evaluation, innovation, policy analysis.

I confirm that I have read this submission and believe that I have an appropriate level of expertise to confirm that it is of an acceptable scientific standard.

# Peer review: Reviewer response for version 1

**DOI:** 10.21956/openreseurope.14692.r27372

**Published:** 2021-08-24

**Authors:** Gernot Pruschak

**Affiliations:** 1 Business School, Bern University of Applied Sciences, Bern, Switzerland

Dear authors,

 Thank you for giving me the opportunity to review your manuscript entitled “Measuring the impact of publicly funded open innovation programs: the case of Data Market Services Accelerator.” This research topic is highly relevant due to the strong increase in publicly funded open innovation initiatives. Thus, it is of utmost importance to generate standard measurement criteria to assess their effectiveness and efficiency. In addition, I appreciate the idea of employing the Data Market Services Accelerator as a case study to test the implementation of the metrics.

 However, whereas the research idea is great, there exist room for improvements in the research execution. I outline my concerns below:

**Standardized measurement scale:**

 The authors` “intention in this article is to identify a flexible and generalizable framework” for measuring the impact of publicly funded open innovation initiatives (p. 7). Yet the results as exhibited in Tables 4 through 7 solely highlight criteria relevant for the DMS. Based on the claims in the introduction (and also the lengthy elaborations on why open data requires standardization), I was surprised to not see a list of criteria applicable to various open innovation programs as the result. In fact, the results part contains the findings from the measures for the DMS. Consequently, there exist two ways forwards: Either the authors reduce their claims in the introduction and transparently depict that the manuscript focuses on reporting the effectiveness and efficiency of the DMS or the authors tone down the DMS related work but instead present the general toolkit as their main results.

**Relevance and coherence of arguments:**

 The manuscript includes (especially in the introduction but also in other parts) lengthy elaborations on topics only peripherally related to measuring open innovation programs. In turn, at other parts the authors jump from one thought to another without providing logical connections. For example, the sub-chapter “Open innovation programmes for startups and SMEs” does not include any example of open innovation programmes (Kindly note that this only represents one of multiple appearances of this problem). Instead, it elaborates in general what open innovation is and how it can be seen in the context of AI. To overcome this issue, I would suggest the authors to rework the whole manuscript and cut all paragraphs that do not directly relate to the main theme. This especially applies to the introduction which is considerably long and contains several paragraphs which are not necessary from a scientific point of view but instead are written in similar fashion with consulting reports which must contain certain words (AI, standardization, Big Data, …) and phrases. On this note, I would also like to remind the authors that good scientific practice implies that we include all information sources as citation. In fact, the first two pages of the manuscript contain only four citations. There exist numerous paragraphs with statements including important contents that do not include references. To make this more comprehensible for readers please include citations for every idea that was not discovered by yourself. As an approximate guideline I would like to give you the following advice: Every sentence containing a statement (not based on your research) that is more specific than “the sky is blue” should include a citation

**Accelerator, incubator and open innovation:**

 “Support programmes for startups and SMEs have typically taken the form of accelerators and incubators. More recently, open innovation programmes have emerged at the intersection of these two paradigms” (p. 2). Whereas the introduction states that this research focuses on the intersection and interplay of accelerators and incubators, the methods and results sections do only focus on accelerator. Again, please ensure coherency throughout the manuscript. On this note, I also doubt that the developed measures can apply to all open innovation programs as open innovation does not only take place within startups and SMEs but also includes stake holders coming from large industry, universities and other research institutions. Consequently, I would suggest toning down the focus on open innovation and instead rely on a more nuanced approach towards entrepreneurship.

**Methods:**

 The survey sample consists of six respondents for the first cohort and 20 respondents for the second cohort. Nevertheless, the authors argue as if the sample size would be sufficiently large by employing percentage numbers and inducing from the survey results that the DMS worked successful. Being a quantitative researcher myself, I strongly doubt the expressiveness of these two surveys. Consequently, I would advise to refrain from using percentages but instead including the actual numbers (e.g. 2 companies instead of 33.3% of our respondents) to transparently depict the small sample size. Moreover, please refrain, especially in the discussion section, from making strong claims based on the survey and workshop results.

**Discussion:**

 The discussion section does not include limitations and suggestion for future research. Please add them. Moreover, readers might want to know the theoretical and scientific implications of your work. In addition, the discussion section should not present or discuss new results. Therefore, I would suggest moving those parts of the “Increasing engagement” sub-chapter highlighting new results (e.g. results from the first workshop) to the results section. Last, the authors argue in the beginning of the discussion that their research shows differences in regard to geographic origins and industry sectors. However, the results section does not split the findings into geographical regions or industry. Please only include elaborations on findings in the discussion section that were also shown in the results section.

 Summing up, I agree with the importance and the potential of the research topic. Yet I believe the manuscript requires substantial improvement work before it can be considered for indexing. Therefore, my suggestion would be a major revision.

If applicable, is the statistical analysis and its interpretation appropriate?

No

Is the study design appropriate and is the work technically sound?

Partly

Is the work clearly and accurately presented and does it engage with the current literature?

No

Are the conclusions drawn adequately supported by the results?

No

Are sufficient details of methods and analysis provided to allow replication by others?

Partly

Are all the source data and materials underlying the results available?

Yes

Reviewer Expertise:

Open Innovation, Open Science, Open Data, Quantitative Research, Surveys, Descriptive Statistics, Parametric Statistics

I confirm that I have read this submission and believe that I have an appropriate level of expertise to state that I do not consider it to be of an acceptable scientific standard, for reasons outlined above.

# Response

**Published:** 2022-04-04

**Authors:** Maria Priestley

**Affiliations:** 21503, UK

Dear Dr. Pruschak,

 We would like to thank you for the detailed feedback which you provided on our manuscript last year. Over the past few months, we have conducted a major revision of the paper in response to your suggestions.

 A full description of the changes can be found in a separate document linked here.

 The main changes can be summarised as follows:

- Our claims about the generalisability of the framework and the strength of claims regarding our results have been reduced. We have also highlighted that our manuscript focuses on measuring the performance of only one programme – the DMS Accelerator.
- More references have been added to support our claims, particularly in the Introduction section.
- We have provided more examples of open innovation, and have justified our decision to describe DMS and other similar initiatives as examples of open innovation, rather than relying on traditional notions of acceleration and incubation. We have also clarified that the scope of our paper focuses on initiatives that serve startups and SMEs, which are only some of the various possible stakeholders that can be targeted by open innovation initiatives.
- We have decided to retain information about the characteristics of data-centric programmes (including words such as “AI”, “standardisation” and “big data”), as these concepts are an essential part of the specialised technical training and advisory services offered by data-centric open innovation programmes. By including these terms in the article, we are able to engage with audiences outside academia who work in data-oriented initiatives.
- We have obtained additional data, which has increased our survey sample size from 20 to 47 respondents across the three DMS cohorts. We have also reported the new results using actual numbers (rather than percentages) for transparency.
- We have now added a section on the “Limitations and future work”.

Once again, thank you for taking the time to consider our submission. Your insights are greatly appreciated. We hope that you will find the updated article more rigorous and worthy of indexing.

# Peer review: Reviewer response for version 1

**DOI:** 10.21956/openreseurope.14692.r27403

**Published:** 2021-08-19

**Authors:** Francesco Mureddu

**Affiliations:** 1 The Lisbon Council, Brussels, Belgium

The present article discusses the question of how impact can be assessed in open innovation programmes related to data and AI. Drawing on the authors’ experience of publicly-funded data incubators in Europe, they review previous approaches to impact assessment, their position in the wider economic context, and what distinguishes data and AI programmes from other types of accelerators in the European tech scene. In that regard, the authors propose a methodology to assess their impact and apply it to one such programme, Data Market Services (DMS). The methodology presented here is being used to monitor and adjust the governance of our services based on work done in the first two years of the programme.

 The paper is very interesting and informative, rigorous and actually very useful also for my professional activity. It is flawless, and I recommend indexing as it is.

If applicable, is the statistical analysis and its interpretation appropriate?

Yes

Is the study design appropriate and is the work technically sound?

Yes

Is the work clearly and accurately presented and does it engage with the current literature?

Yes

Are the conclusions drawn adequately supported by the results?

Yes

Are sufficient details of methods and analysis provided to allow replication by others?

Yes

Are all the source data and materials underlying the results available?

Yes

Reviewer Expertise:

Impact assessment and evaluation, innovation, policy analysis.

I confirm that I have read this submission and believe that I have an appropriate level of expertise to confirm that it is of an acceptable scientific standard.

# Response

**Published:** 2022-04-04

**Authors:** Maria Priestley

**Affiliations:** 21503, UK

Dear Dr. Mureddu,

 We would like to thank you very much for your encouraging feedback on our manuscript last year. We wanted to let you know that over the past few months we have made some revisions to the article in response to the second reviewer’s comments.

 A full description of the changes can be found in a separate document linked here.

 The main changes can be summarised as follows:

- Our claims about the generalisability of the framework and the strength of our claims regarding the results have been reduced. We have also highlighted that our manuscript focuses on measuring the performance of only one programme – the DMS Accelerator.
- More references have been added to support our claims, particularly in the Introduction section.
- We have provided more examples of open innovation, and have justified our decision to describe DMS and other similar initiatives as examples of open innovation, rather than relying on traditional notions of acceleration and incubation. We have also clarified that the scope of our paper focuses on initiatives that serve startups and SMEs, which are only some of the various possible stakeholders that can be targeted by open innovation initiatives.
- We have obtained additional data, which has increased our survey sample size from 20 to 47 respondents across the three DMS cohorts. We have also reported the new results using actual numbers (rather than percentages) for transparency.
- We have now added a section on the “Limitations and future work”.

 Once again, thank you very much for taking the time to consider our submission. We hope that the updated version still resonates with your experience and remains useful to your work.
